# Dark spot detection for characterization of marine surface slicks using UAVSAR quad-pol data

**DOI:** 10.1038/s41598-021-88301-9

**Published:** 2021-04-26

**Authors:** Vaishali Chaudhary, Shashi Kumar

**Affiliations:** Photogrammetry and Remote Sensing Department, Indian Institute of Remote Sensing (ISRO), Dehradun, India

**Keywords:** Natural hazards, Environmental social sciences

## Abstract

Oil spills are a potential hazard, causing the deaths of millions of aquatic animals and this leaves a calamitous effect on the marine ecosystem. This research focuses on evaluating the potential of polarimetric parameters in discriminating the oil slick from water and also possible thicker/thinner zones within the slick. For this purpose, L-band UAVSAR quad-pol data of the Gulf of Mexico region is exploited. A total number of 19 polarimetric parameters are examined to study their behavior and ability in distinguishing oil slick from water and its own less or more oil accumulated zones. The simulation of compact-pol data from UAVSAR quad-pol data is carried out which has shown good performance in detection and discrimination of oil slick from water. To know the extent of separation between oil and water classes, a statistical separability analysis is carried out. The outcomes of each polarimetric parameter from separability analysis are then quantified with the radial basis function (RBF) supervised Support Vector Machine classifier followed with an accurate estimation of the results. Moreover, a comparison of the achieved and estimated accuracy has shown a significant drop in accuracy values. It has been observed that the highest accuracy is given by LHV compact-pol decomposition and coherency matrix with a classification accuracy of ~ 94.09% and ~ 94.60%, respectively. The proposed methodology has performed well in discriminating the oil slick by utilizing UAVSAR dataset for both quad-pol and compact-pol simulation.

## Introduction

**Figure 1:**
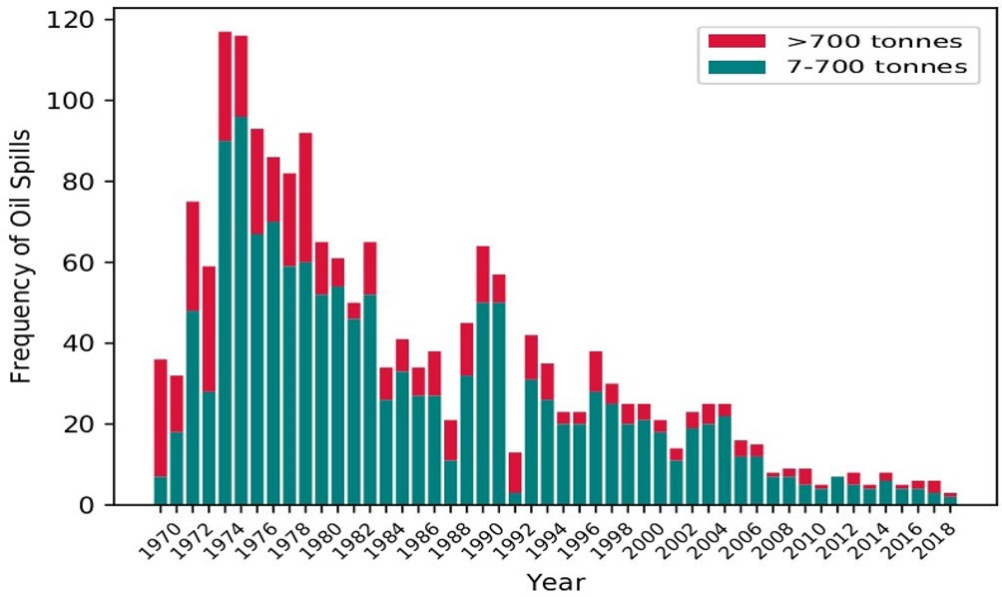
Oil spill occurrences from tanker incidents worldwide from 1970−2010 (Source: (ITOPF, 2020)).

Marine slicks are a serious threat to the aquatic ecosystem and the wildlife relying on them. A report from International Tanker Owners Pollution Federation Ltd (ITOPF) reported about 5.73 million tonnes of oil released into the waters solely because of the accidents of tankers between 1970 and 2016^1^. Some of the oil spill incidents have occurred on such a large scale that they were registered amongst the most disastrous oil spill incident in history. The five biggest oil spills in history are: The Lakeview Gusher, California (1910–1911)^2, 3^, oil spill of The Gulf War (1991)^4^, The Deepwater Horizon (2010)^5^, Ixtoc I oil spill, Mexico (1979–1980)^6^, and The Niger Delta’s enduring oil spill (1976 − 1996)^7^. A rough estimation of oil entering into the water is considered around 1,300,000 metric tonnes per year^8^ making it one of the most serious threats to the marine ecosystem and the surroundings. The contribution of natural seepage in overall oil spills incidents has been increased from 3,00,000 to 6,00,000 tonnes/year and this amount sums for approximately 46% of the total oil spill occurrences^9^ (reported in the Joint Group of Experts on the Scientific Aspects of Marine Environmental Protection (GESAMP) in 2007). The origin of natural seeps lies beneath the sea bed in the geological strata. According to the National Research Council report in 2003, the natural seepage shares a large percentage of the overall number of spills volume even after having a low occurring frequency. Figure 1 shows the variation in the occurrence of oil spill incidents over a five-decade timespan. The number of incidents with oil spill volume larger than 700 tonnes is very low compared to the incidents with oil volume between 7 to 700 tonnes. According to the International Tanker Owners Pollution Federation (ITOPF) data, the number of bigger oil spill incidents decreased from 24.5 (in 1970) to 1.7 oil spills per year till 2010^1, 10, 11^.

A peak in oil spill occurrences can be seen between 1974−1975, with the year 1974 having the highest number of occurrences (see Fig. 1). Even after the decrement in the frequency of large oil spill incidents, they still have negative impacts on the ecosystem as the oil may disperse deep into the water, lie on the seafloor for many years, and require consistent surveillance and remote monitoring. The advancement in monitoring these remote areas has grown significantly by using remote sensing techniques with the help of airborne and spaceborne platforms. Both the optical and microwave monitoring options are exploited widely for detection of the oil spill incidents. However, discrimination between water and marine slick is considered a difficult task in optical data as the oil slick exhibits a very small degree of optical properties to be detected. Although there are a few studies about exploring the optical data potential for oil slick mapping and change detection^12, 13^. In microwave remote sensing, airborne Synthetic Aperture Radar (SAR) systems are being widely used for their fast responses and low operating costs. UAVSAR is one such airborne SAR platform for monitoring the areas of interest using microwaves of different wavelengths. It has provided great results in oil slick identification and short-time repetitive monitoring. With controlled parameters and altitude, UAVSAR can survey the regions anytime without any atmospheric dependency and provide high-resolution imagery with a high Signal to Noise Ratio (SNR).

UAVSAR L-band sensor has been proved very efficient in tracking the marine slicks with 9–12 m/s wind conditions for a duration of 8 h^14^. The radar backscatter from the oil-covered area depends highly on the roughness of the sea surface. The oil slick has viscoelastic properties which dampen the small gravity waves and capillary waves due to subsequent decrement in wind friction over the oil slick surface and reduction in surface tension, resulting in wave dissolution^15^. Moreover, other features like rain cells, low wind zones, calm sea zones, natural seeps, etc.show similar backscattering responses as oil spills. This change in contrast (between water and possible oil spill) is not consistent in the SAR image due to the complex underlying architecture of SAR^16^. Hence precise identification of oil slicks in water bodies needs a timely response for executing the countermeasures.

Many oil spill studies have been carried out in past using SAR technology^17–24^ by applying several techniques including adaptive thresholding^25^, using a genetic algorithm of artificial intelligence approach followed by receiver-operating characteristics for validation^26^. Whereas^27^ has shown the discrimination between the water and oil slick by analyzing the conformity coefficient successfully for the water body and oil slick using RADARSAT-2 sensor data. A self-similarity parameter was introduced to discriminate between the oil spill and look-alikes using UAVSAR and RADARSAT-2 SAR data followed by the Random forest classification technique^28^. The effectual Noise Equivalent Sigma Zero (NESZ) merits of UAVSAR L-band sensor provide good oil slick discrimination potentiality^29^ as the low noise floor in SAR is very effective in detecting the oil slick areas^30^.

A novel approach using simple Otsu segmentation followed by the neural network classifier (a Back Propagation Network model) was used by^31^ to successfully discriminate between the oil spills and look-alikes. In a recent study, spaceborne and airborne SAR data has been utilized for successful oil spill mapping, using separability analysis, decomposition models, and supervised classification techniques^32^. Oil spills were also detected using dual-threshold segmentation and using the Support Vector Machine to classify the results^33^. One of the main reasons behind SAR sensor’s potential in identifying the targets is its capability to discriminate the target signatures from each other based on the underlying scattering mechanisms^34^. Each data type has different statistical properties hence it becomes vital to build a robust system for oil spill discrimination. Similarly^35^, utilized a Multi-Source Image Processing System which provided a suitable statistical model for each data type and their stochastic distances to differentiate sheen from the thicker layer.

Several polarimetric parameters have been analyzed frequently for oil spill detection and characterization. Some of the most utilized parameters have been jotted down in Table 1 along with the methodology followed and SAR sensor for the respective study. It is noteworthy that Entropy, Anisotropy, Scattering angle, and VV-damping ratio are some of the frequently used polarimetric parameters. The proposed study utilized some of these features to examine their potential in distinguishing the zones within the oil slick based on the backscattering. Moreover, derived Compact-pol LHV and RHV components have provided good results compared to other features tested in this study (refer to “Results and discussion”).

**Table 1:** Most frequently used polarimetric parameters in the literature for oil spill detection.

Author	Methodology/parameters used	Sensor
^36^	Entropy and Anisotropy based characterization followed by SVM classification	RADARSAT-2
^22^	Entropy, Anisotropy, scattering angle, and a combination of these as n new parameter F followed by Otsu segmentation	UAVSAR
^37^	Entropy (H), Scattering Angle (α), Pedestal Height (PH), Conformity Coefficient, Degree of Polarization, Std. Co-Polarized Phase Difference, Correlation Coefficient, Coherence Coefficient, Ellipticity	RADARSAT-2
^38^	Damping ratio and co-polarization ratio	UAVSAR
^39^	Entropy and damping ratio	RADARSAT-2, TerraSAR-X, UAVSAR
^40^	Damping ratio	UAVSAR
^41^	Conformity coefficient for finding out the dominant scattering and discriminating between surface, double-bounce, and volume scattering	RADARSAT-2
^42^	Copolarization power ratio, Geometric intensity, Copolarization cross product, Standard deviation of co-polarized phase difference, Entropy, Anisotropy	RADARSAT-2

**Figure 2:**
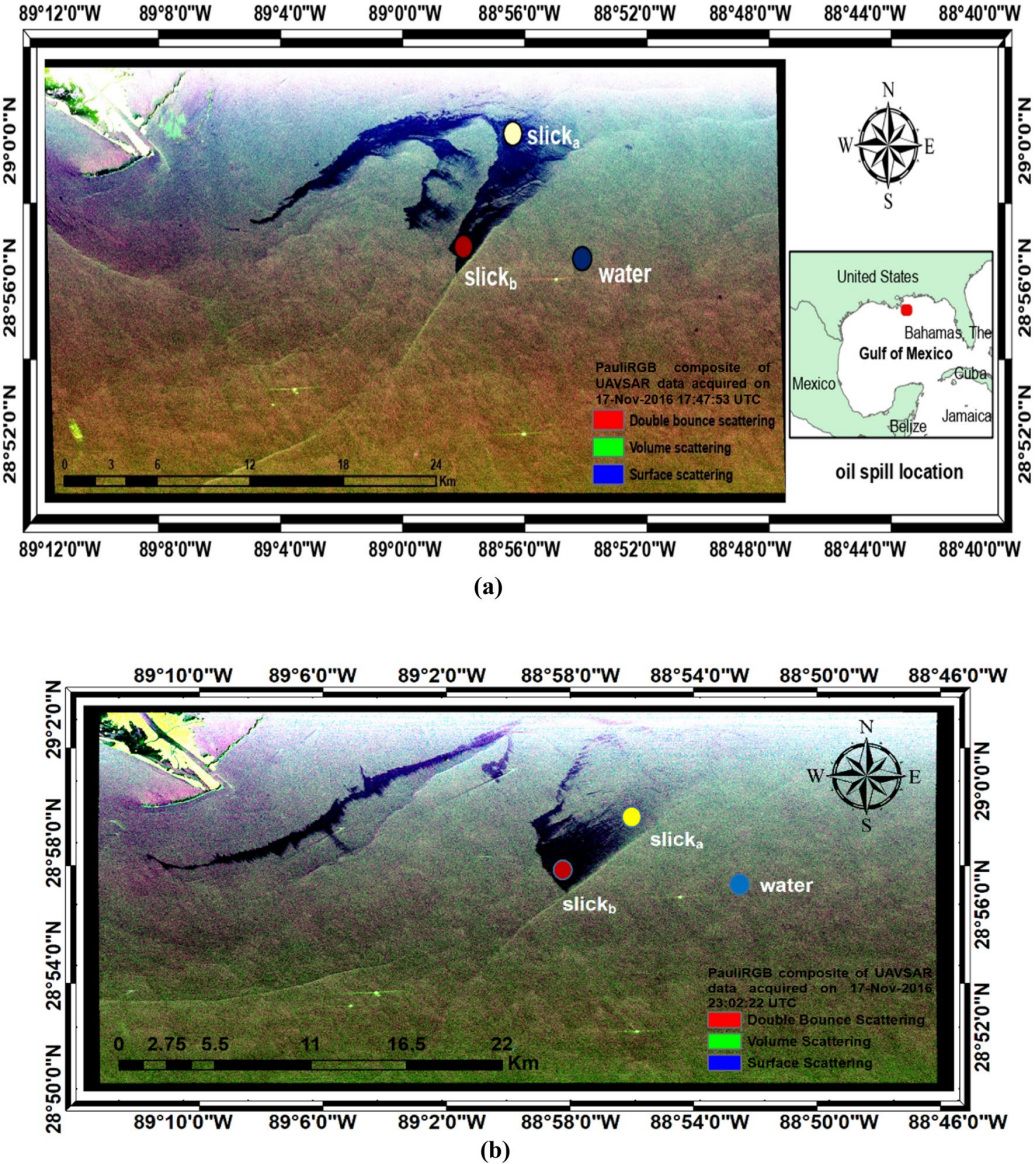
(**a**) Pauli RGB representation of DATA1 and the oil spill location (Gulf of Mexico), (**b**) Pauli RGB representation of the DATA2 (Gulf of Mexico)^*^. ^*^Maps were created using ArcMap software by Esri. Please visit www.esri.com or https://support.esri.com/en/products/desktop/arcgis-desktop/arcmap/10-6.

The prime focus of the proposed study is to analyze the capability of different polarimetric parameters in detecting the variation of backscattering intensity within the oil spill region from the possible thick and thin (sheen) oil slick zones (shown in Fig. 2). A significant effect of weathering can be seen in some areas of the slick. These areas have a relatively fainter (or less dark) signature than the areas with more accumulation of oil. These zones with more amount of oil within the oil slick are created due to the effect of wind or water current. This study is based on the difference in backscattering between sheen and the region with more oil accumulation. Hence, the oil slick was hypothetically divided into two regions namely, region 1: slick_*a*_ and region 2: slick_*b*_. slick_*a*_ appears a few degrees faded in comparison to slick_*b*_. It is important to note here that these name conventions are just to make the interpretations easy and this does not validate the outcomes.

## Materials and methodology

### Study area and data

The ship activities in the gulf area make the nearby area quite susceptible to oil spillages. In 2004, hurricane Ivan caused hit the Gulf of Mexico and damaged the Mississippi Canyon-20 oil rig which was operated by Taylor Energy^43^. The platform sank in September 2004 but it took a whole decade to locate the oil rig and till then the oil kept leaking. An approximation of oil leakage between 9 to 108 barrels per day was speculated in a 2019 report by National Oceanic and Atmospheric Administration (NOAA)^44^.

UAVSAR L-band fully polarimetric data of this region is chosen for this study. Two UAVSAR datasets are utilized for testing the methodology. Both the datasets are of the same region and same date but both are captured at a different time with different oil slick trajectories. The PauliRGB representation for both datasets has been provided in Fig. 2. The DATA1 and DATA2 are already radiometrically calibrated with an approximate incidence angle range of 21.45° to 65.37° and 21.45° to 65.38° respectively. All the necessary details can be found in Table 2.

**Table 2:** Dataset details and properties.

Parameters	DATA1	DATA2
Product Id	gulfco_27086_16100_008_161117_L090_CX_01	gulfco_27086_16101_003_161117_L090_CX_01
Product type	Ground range projected (equiangular) and multi-looked data	Ground range projected (equiangular) and multi-looked data
Date and time of acquisition	17-Nov-2016 17:47:53 UTC	17-Nov-2016 23:02:22 UTC
Number looks in range and Azimuth	3, 12	
Acquisition mode	PolSAR	
Bandwidth	80 MHz	
No. of scan lines and samples	3751, 7738	3751, 7790

### Methodology

**Figure 3:**
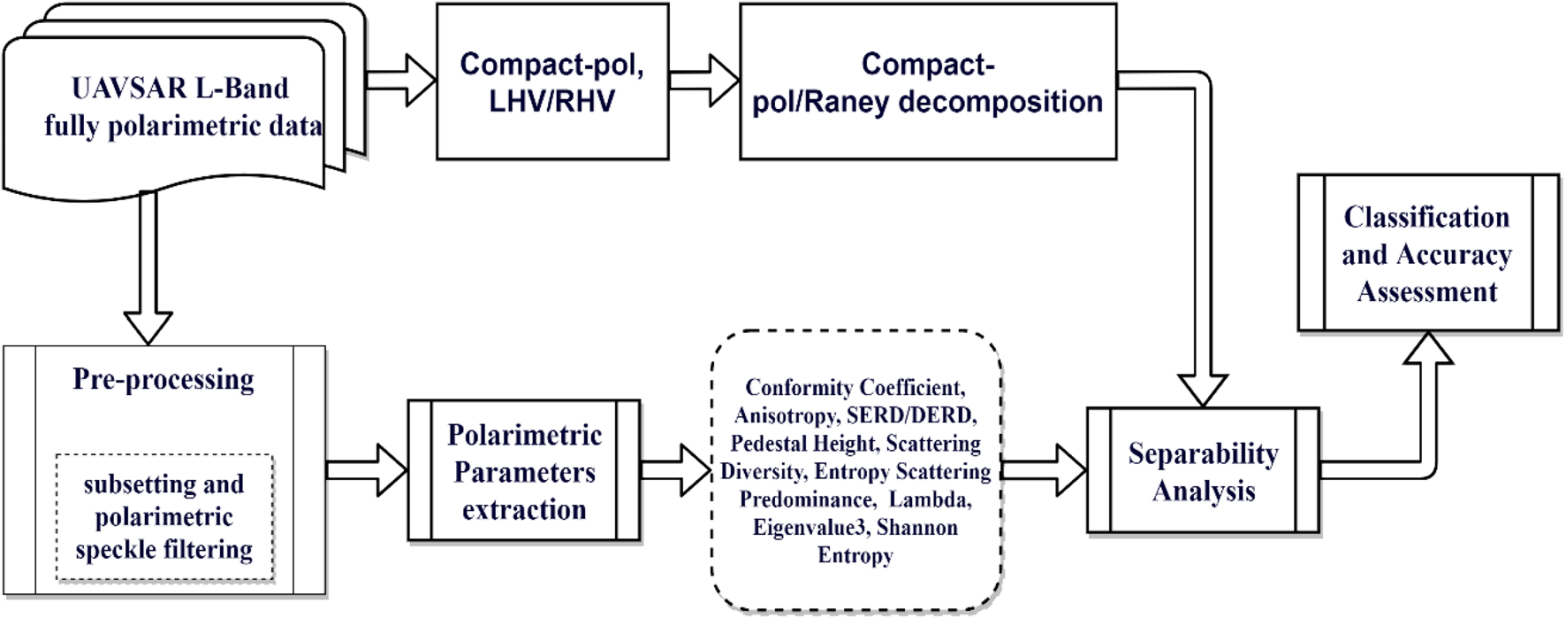
Flowchart of the proposed methodology for the study.

The polarimetric properties of the SAR sensor make it an excellent instrument to analyze various scattering mechanisms occurring in the region of interest. Several studies have analyzed the oil slick properties using the polarimetric characteristics of the SAR sensor^45^, extracting the polarimetric features, combining them with traditional parameters^46^, and generating new features^47^. The followed methodology for this study has been presented in Fig. 3.

**Figure 4:**
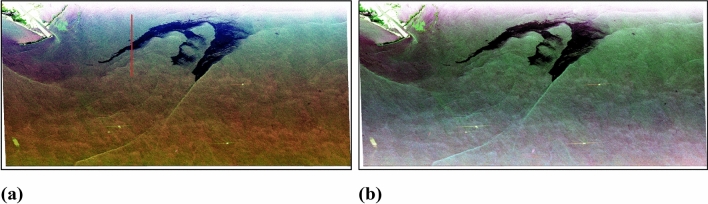
Figures showing Pauli RGB composite of data (**a**) before incidence angle correction, and (**b**) after incidence angle correction at reference angle ${\theta }_{ref}$= 43.42636.

Since UAVSAR is an airborne platform, it covers a range swath of 16 km at a very low altitude of approximately 13,800 m. At this altitude, UAVSAR system captures data with a range of incidence angles from near to far range. And due to this, sometimes significant changes in backscattering intensity can be observed varying from near to far range. To remove this pattern, incidence angle correction was implemented on the dataset. Cosine correction is one of the formerly developed and frequently used incidence angle normalization techniques^48–50^. The datasets before and after the incidence angle correction are presented in Fig. 4. The details about the incidence angle correction procedure can be found in appendix section A.1.

Almost every SAR data suffers from coherent interference within the received signals which results in the formation of the salt and pepper effect, briefly known as speckle^51, 52^. The presence of speckle in the image may result in poor classification outcomes. To improve this coarse texture of the imagery, Refined Lee, a polarimetric speckle filter with a window size 3 × 3 has been applied to the dataset. In a study, the author^53^ has studied the effect of noise floor on various parameters for oil slick detection, using a receiver operating characteristic curve. This study reviews 19 polarimetric parameters for the successful extraction of oil spills. These calculated parameters are analyzed for the degree of separation between oil and water. The ability to discriminate between potential thin−thicker and water regions is exploited for each parameter followed by the supervised Support Vector Machine (SVM) classification. Details about the utilized polarimetric parameters are stated in “Polarimetric parameters”.

### Polarimetric parameters

The polarimetric SAR data has the potential to retrieve scattering-based information for several parameters for oil slick detection^22, 54–56^. A brief description of the parameters utilized for this study to discriminate between water–oil and potential thin-thick regions within the oil slick has been provided in the following subsections.

#### Conformity coefficient

The Conformity Coefficient $\left(\mu \right)$ was first derived for the soil moisture inversion using compact-polarimetric data^57^. The parameter proves itself very useful in discriminating the surface scattering, double-bounce scattering, and volume scattering in the region of interest. Equation 1 gives the mathematical notation of $\mu$.

1 $$\mu \approx \frac{2\left[Re\left({S}_{HH}{S}_{VV}^{*}\right)-{\left|{S}_{HV}\right|}^{2}\right]}{\left({\left|{S}_{HH}\right|}^{2}+2{\left|{S}_{HV}\right|}^{2}+{\left|{S}_{VV}\right|}^{2}\right)}$$

If ${S}_{HV}$ is close to 0 then ${S}_{HH}$ and ${S}_{VV}$ are highly correlated with phase difference close to $0$ resulting in $\mu \ge 0$ (in the case of Bragg’s scattering). On the other hand, for the Non-Bragg scattering mechanisms (for e,g., vessels) ${S}_{HH}$ and ${S}_{VV}$ are weakly correlated resulting in $\mu <0$^27^. $\mu$ has been utilized for oil spill detection showing negative values of oil slick area with RADARSAT-2 dataset^27^ but the value of $\mu$ highly depends upon the dataset noise floor^41^ hence it tends to give positive values for both the oil spill and the water region.

#### Scattering diversity

The scattering diversity was proposed by^58^ as an alternative to the entropy parameter as it is highly correlated with entropy. The mathematical interpretation of scattering diversity is given in Eq. (2).

2 $$\widehat{H }=\frac{3}{2}\left(1-{\Vert N\Vert }_{F}^{2}\right)$$

The number of scattering mechanisms is indicated by calculating the reciprocal of the Frobenius norm of $N$. Scattering diversity has been utilized earlier for the oil slick detection using an Artificial Neural Network framework with RADARSAT-2 and TerraSAR-X datasets^59^. Scattering diversity can help in detecting the partially or depolarized signal from the target as it has a direct relationship with depolarization^58^.

#### SERD/DERD

Single-Bounce Eigenvalue Relative Difference (SERD) and Double-Bounce Eigenvalue Relative Difference (DERD) are the eigenvalues-based parameters proposed by^60^. The basis of SERD is the eigenvalue and eigenvector-based decomposition proposed by^61^. The parameter compares the relative importance of different scattering mechanisms and becomes important with data having large entropy values. SERD is considered quite sensitive to surface roughness. The oil spills make the surface smooth and possess high entropy but this is not always true as entropy is affected by NESZ level. High values of entropy indicate the presence of different scattering mechanisms, hence SERD becomes relatively small, and vice-versa. On the other hand, DERD exhibits similar behavior like Anisotropy (A) for the low roughness values but it works differently for high-frequency values, see Eq. (3).

3 $$SERD=\frac{{\lambda }_{s}-{\lambda }_{{3}_{nos}}}{{\lambda }_{s}+{\lambda }_{{3}_{nos}}} \quad DERD= \frac{{\lambda }_{d}-{\lambda }_{{3}_{nos}}}{{\lambda }_{d}+{\lambda }_{{3}_{nos}}}$$

where ${\lambda }_{s}$ and ${\lambda }_{d}$ are the eigenvalues associated with the single- and double-bounce scattering behavior, respectively.

#### Shannon entropy

Shannon entropy estimates the degree of uncertainty of the random variables. It has low values for quasi-deterministic random variables and large values for oscillating variables^62^. In polarimetric terms, for a radar illuminated medium, Shannon entropy statistically calculates the disorganization of the medium^63^. The Shannon entropy of a random variable $X({X}_{1},{X}_{2},\dots {X}_{n})$ having probabilities (${p}_{1},{p}_{2},\dots {p}_{n}$) is calculated as given in Eq. (4).

4 $$E\left(X\right)=\sum_{i=1}^{n}\left[{p}_{i}\mathrm{log}\left(\frac{1}{{p}_{i}}\right)\right]$$

In Shannon entropy, oil slick possesses a high degree of randomness and low span values. The value of the oil slick (dark regions in microwave sensor imagery) areas shows comparatively lower values than the water region^64^.

#### Pedestal height

The amount of variation in the scattering properties is termed Pedestal height. It gives the estimation of the degree of polarization of the returned signal (see Eq. 5). The value of pedestal height varies proportionally to the degree of depolarization i.e., a high value of Pedestal height will indicate a high degree of depolarization while the low values of Pedestal height show the dominance of surface scattering^65^.

5 $$Pedestal \, Height=\frac{\mathrm{min}({\lambda }_{1},{\lambda }_{2},{\lambda }_{3})}{\mathrm{max}({\lambda }_{1},{\lambda }_{2},{\lambda }_{3})}$$

Pedestal height is considered a very important factor in detecting the presence of oil over water. The areas covered by oil have a sufficiently large damping effect on water current, hence a large value of Pedestal height is obtained in comparison to the clean sea area^54^.

#### Eigenvalue based polarimetric parameters: H/A/α decomposition

H/A/α decomposition was proposed by^66^. In this decomposition, the generation of the coherency matrix $\left(T3\right)$ is based on the analysis of the eigenvector. The coherency matrix $\left(T3\right)$ is represented in Eq. (6) as:

6 $$T\left(3\right)={U}_{3}\sum {U}_{3}^{*}$$

here ∑ represents a diagonal matrix having $T\left(3\right)$ eigenvalues $\lambda i (i=\mathrm{1,2},3)$, $U$ is the Unitary matrix with the eigenvectors and ${U}^{*}$ is the complex conjugate transpose of $U$^66^. Lambda $(\lambda )$ is a non-negative real eigenvalue in the diagonal matrix. The H/A/α parameters were explained by^66^ as shown in Eqs. (7,8, and 9).

7 $$H=\sum_{i=1}^{3}{p}_{i}{\mathrm{log}}_{3}\left({p}_{i}\right); \quad {\mathrm{ where }}\,{p}_{i}=\frac{{\lambda }_{i}}{{\lambda }_{1}+{\lambda }_{2}+{\lambda }_{3}}$$

8 $$A=\frac{{\lambda }_{2}-{\lambda }_{3}}{{\lambda }_{2}+{\lambda }_{3}}$$

9 $$\alpha =\sum_{i=1}^{3}{p}_{i}{\alpha }_{i}$$

All three parameters i.e., Anisotropy $(A)$, Entropy $(H)$, and Alpha ($\alpha$) have great physical significance in mapping oil slicks. The sea surface possesses a low degree of $H$ as it has dominant single scattering. The presence of oil slick on water increases the $H$ value. Similarly, a low value of $\alpha$ angle shows the existence of a single dominant scatterer. Whereas, $A$ represents the probability of scattering dominance between the second and third eigenvalue^21, 47^. The value of $A$ can be $0$ for rough surfaces and its values greater than $0$ indicates the existence of multiple scatterers^67^. Ideally, $A$ possess typically high values for the oil-covered region in comparison to the oil-free area.

The eigenvalues of a coherency matrix are related to the surface roughness. This relationship exhibits a physical significance related to the scattering amplitudes. Hence the rough and smooth surfaces can be generalized in the form of the ratio of the amplitudes^67^. Moreover, ${\lambda }_{1}$ to corresponds to dominant scattering ${\lambda }_{2}$ corresponds to second scattering and ${\lambda }_{3}$ corresponds to third scattering (depolarization caused by the media randomness)^60^.

### Damping ratio and Co-polarization power ratio (CPR)

SAR has provided very reliable results in detecting oil slicks, especially with UAVSAR, as it has a comparatively low noise floor. VV intensity in airborne parameters has been proved to be a great parameter in detecting oil emulsions with reduced dependency on incidence angle. Using the VV channel, the damping ratio provides good results in detecting oil slick over the water surface. Some previous literature has shown successful detection of oil slick based on its thickness using VV damping ratio^68–70^. A clean water surface without oil has a damping ratio near 1 but the presence of oil over the water increases this ratio as the oil dampens the capillary and short gravity waves. The Damping Ratio is defined as the intensity contrast between the oil-free and oil-covered water surface. Mathematically VV damping ratio is given as:

10 $${Damping\,Ratio}_{VV}=\frac{{VV}_{clean sea}}{VV}$$

**Figure 5:**
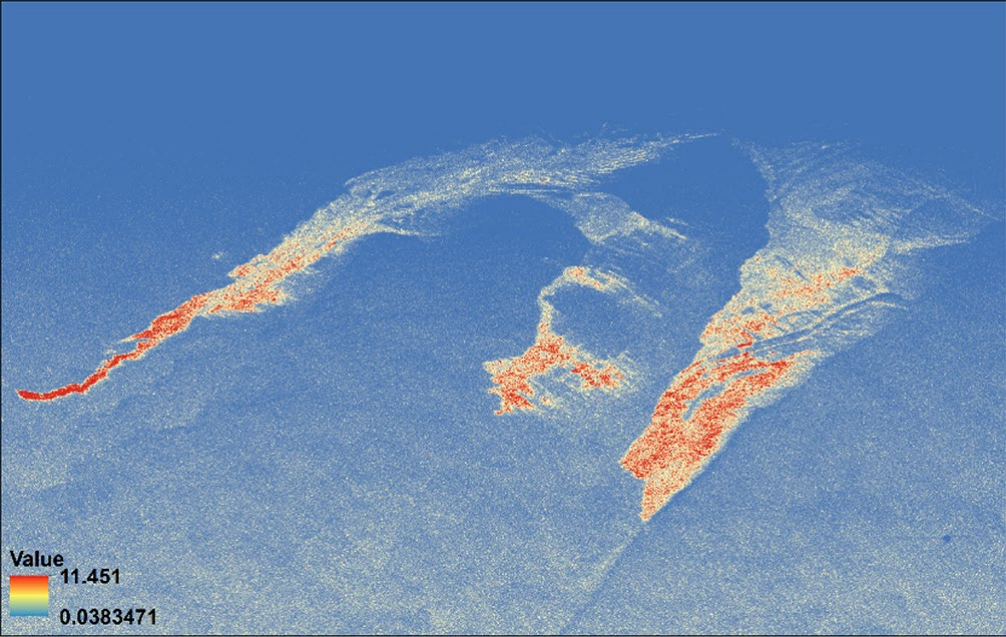
VV damping ratio of the UAVSAR image values ranging from 0.0038 to 11.451.

Here in Eq. (10), ${VV}_{clean sea}$ is the average intensity of a large water surface area with no ships and slick. Using this ${VV}_{clean sea}$ value, the ratio of the full image is done at each pixel position. The yellow color (see Fig. 5) shows the thin oil layers over the water as sheen while the darker red areas within the slick show the accumulation of oil in those zones. The maximum value achieved using the damping ratio is 11.451. A previous study^19^ has used the ${Damping Ratio}_{VV}$ for the same oil slick, also had provided similar outcomes. For example, in the article^40^, the author found that the ${Damping Ratio}_{VV}$ was greater than 8 in the areas where the accumulation of the oil was most prominent and the oil slick was detectable in general with damping ratio values greater than 2.

Another parameter CPR has been investigated in many studies^19–21, 42, 71^ for the detection of marine slicks. Typically, this parameter is independent of the damping effect caused by capillary/ gravity waves in a tilted Bragg model. For the successful detection of oil slick by CPR, there must be a significant change in the dielectric constant (a higher concentration of oil slick should be present). The value of CPR increases on movement from oil-free to oil-covered region. The CPR ratio is mathematically given as Eq. (11),

11 $$CPR=\frac{\langle {\left|{S}_{HH}\right|}^{2}\rangle }{\langle {\left|{S}_{VV}\right|}^{2}\rangle }$$

Since both the damping ratio and CPR give a bright signature of the oil slick, several combinations using these two parameters were tested for improved detection of the oil slick. The combination that gave the most appropriate outcome is stated in Eq. 12.

12 $${Damping Ratio}_{VV}*CPR$$

**Figure 6:**
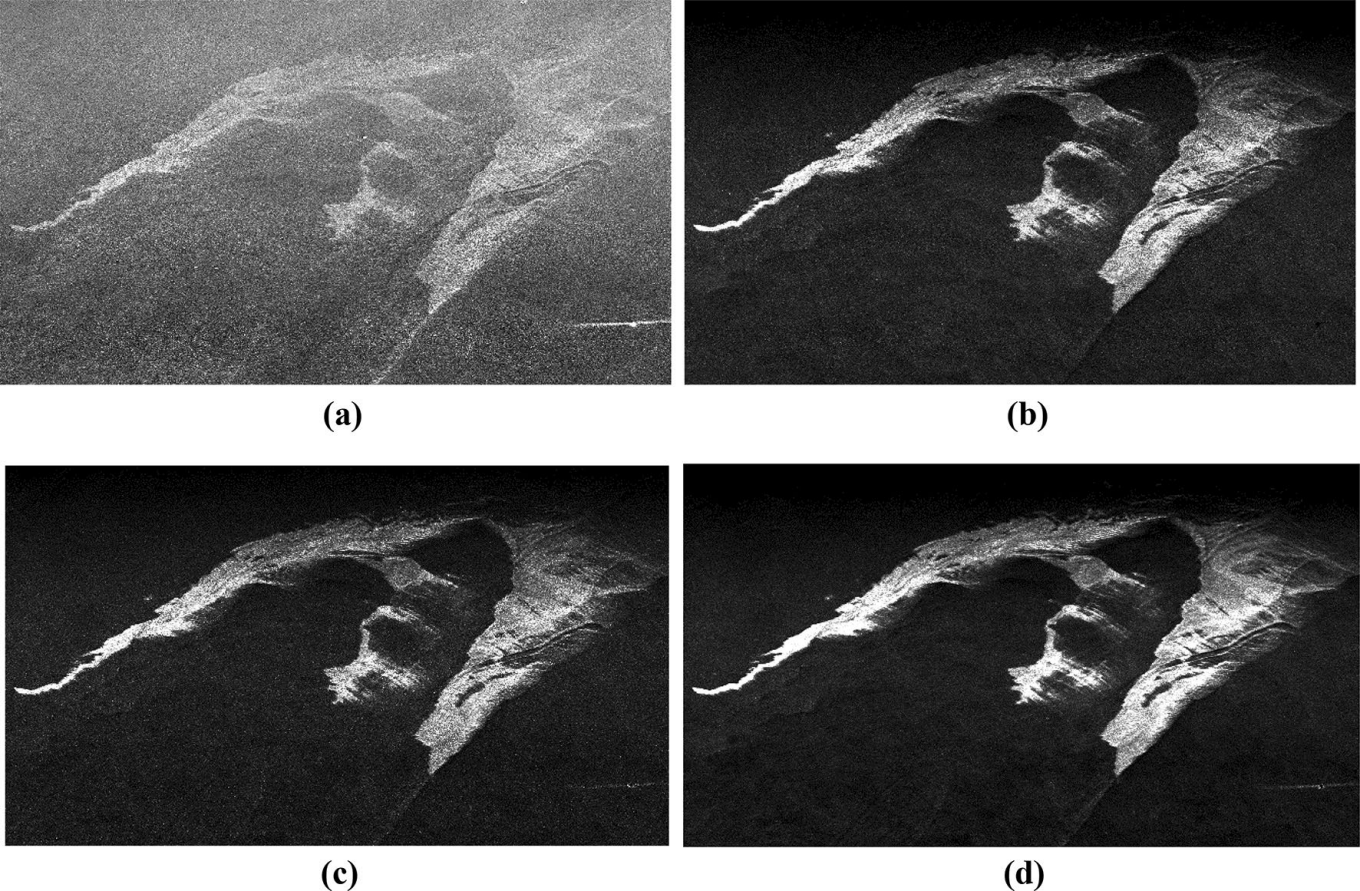
Figure showing parameters (**a**) CPR, (**b**) DR, (**c**) CPR*DR, (d) CPR*DR after applying a 3 × 3 low pass filter.

The calculated image is then passed through a low-pass filter with a window size of 3 × 3 (see Fig. 6). From the resultant image, a comparatively better contrast between the oil slick and the water surface was achieved with relatively less background noise. However, a relative decrement in noise was observed without the application of a low pass filter hence the application of filter enhanced the image more. CPR is considered an important parameter because of its independence of surface roughness, on the other hand, damping ratio depends on the surface roughness. Combining CPR with damping ratio exploits properties of both the parameters by effectively enhancing the oil–water contrast and uniforming the water backscattering up to some extent.

### Simulating compact-pol from quad-pol data

Compact-pol LHV and RHV components are also simulated from the quad-pol data and their potential of extracting the oil slicks from water bodies was also studied. Compact-pol data generally require less system complexity and storage allocation for the data. The main goal of simulating the compact-pol data from quad-pol data was to exploit the full potential of both the data types using only a single dataset. A fully polarimetric SAR data is used as input and the output resulted in a 2 × 2 complex covariance matrix. The author in^72^ has stated this conversion in mathematical interpretations in the case of Single Look Complex (SLC) data where the scattering matrix is projected as given below.

13 $$\left[\begin{array}{c}{E}_{HC}\\ {E}_{VC}\end{array}\right]=\frac{1}{\sqrt{2}}\left[\begin{array}{cc}{S}_{HH}& {S}_{HV}\\ {S}_{VH}& {S}_{VV}\end{array}\right]\left[\begin{array}{c}1\\ \pm i\end{array}\right]=\frac{1}{\sqrt{2}}\left[\begin{array}{c}{S}_{HH}\pm {S}_{HV}\\ {S}_{VH}\pm {iS}_{VV}\end{array}\right]$$

In Eq. (13), the + sign represents the transmit in left-hand circular (LHC) mode, similarly, the—sign represents the transmit in right-hand circular (RHC) mode. For both, LHV and RHV components similar procedure was followed for oil slick detection. Further, Compact-pol decomposition proposed by^72^ and Raney decomposition^73^ was also implemented for both LHV and RHV components. Compact-pol decomposition is a three pseudo-element based decomposition with ${P}_{S}$ element for surface scattering, ${P}_{D}$ is for double-bounce scattering and ${P}_{V}$ represents volume scattering element (see Eq. 14 ).

14 $$\left[\begin{array}{c}{P}_{D}\\ {P}_{V}\\ {P}_{S}\end{array}\right]=\left[\begin{array}{c}\frac{1}{2}{g}_{0}m\left(1-\mathrm{cos}2{\alpha }_{S}\right)\\ {g}_{0}\left(1-m\right)\\ \frac{1}{2}{g}_{0}m\left(1+\mathrm{cos}2{\alpha }_{S}\right)\end{array}\right]$$

On the other hand, Raney decomposition also calculates three elements for surface, dihedral, and volume scattering. Further, supervised SVM classification was applied to both the components and the decomposition results. The SVM classification for both the components has shown high accuracy in detecting and separating one oil slick type from another.

### Support vector machine

The conceptualization of the Support Vector Machine (SVM) was proposed by Vapnik and co-workers^74, 75^ as a learning method with limited or small training samples to provide good classification results. SVM works on the principle of detecting an optimal hyperplane with maximum separation for the distinction of two or more classes in an N-dimensional space along with risk minimization techniques^76^. The basic functionality of SVM is calculating the classification function with the help of the input training data that is fed into the classifier^77^. Ideally, an SVM model consists of three main components: feature selection, support vectors selection, and selecting the kernel functions and related parameters^78^. For this study, the Radial Basis Function (RBF) kernel^79^ was utilized. Here, gamma $(\gamma )$ decides the extent of the kernel spread and determines how far the influence of a single data point can affect the data. A typical representation of SVM based on RBF kernel is given in Eq. (15).

15 $${K}_{RBF}\left(x,{x}^{^{\prime}}\right)=exp\left[-\gamma {\Vert x-{x}^{^{\prime}}\Vert }^{2}\right]$$

The value of RBF kernel depends on the distance from some point/origin. Here $\Vert x-{x}^{^{\prime}}\Vert$ is the Euclidean distance between $x$ and ${x}^{^{\prime}}$. With the help of this distance the similarity index of $x$ and ${x}^{^{\prime}}$ is calculated. Natively, SVM is designed to work on binary classification problems, but for a multiclass problem, SVM utilizes the One-to-One or One-to-Rest approach. Hence, the multiclass problem is broken down into smaller multiple binary problems and the selection of hyperplane is done accordingly between respective classes. The basic algorithm for SVM is given below:
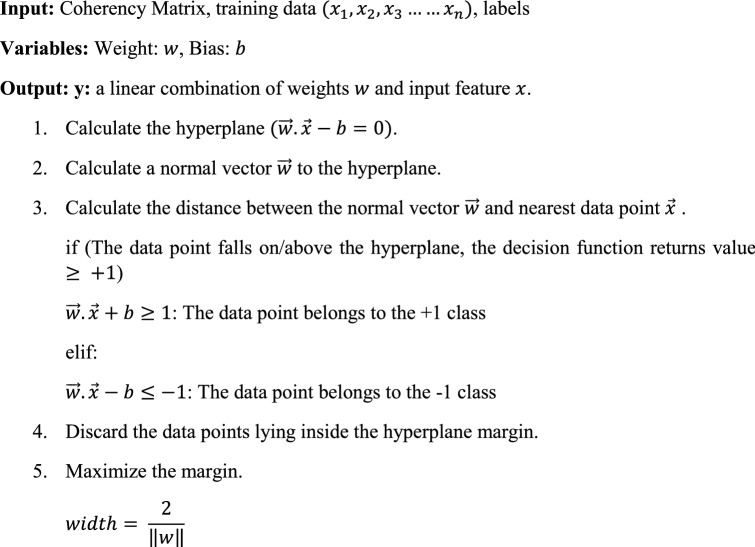


The parameter weight $(w)$ in SVM is the coordinate of the vector orthogonal to the hyperplane. Calculating the dot product of $w$ and feature $x$, the position of the data point can be estimated (step 3-To which class or side the data point belongs). Another parameter bias $(b)$ plays a vital role in maximizing the hyperplane margin. Without bias, the hyperplane will cross through the origin and will eventually fail in achieving the maximum margin (step 5). The SVM algorithm was applied to each parameter including the coherency matrix. The classification of these parameters resulted in the generation of a single classified image for respective parameters.

## Results and discussion

**Figure 7:**
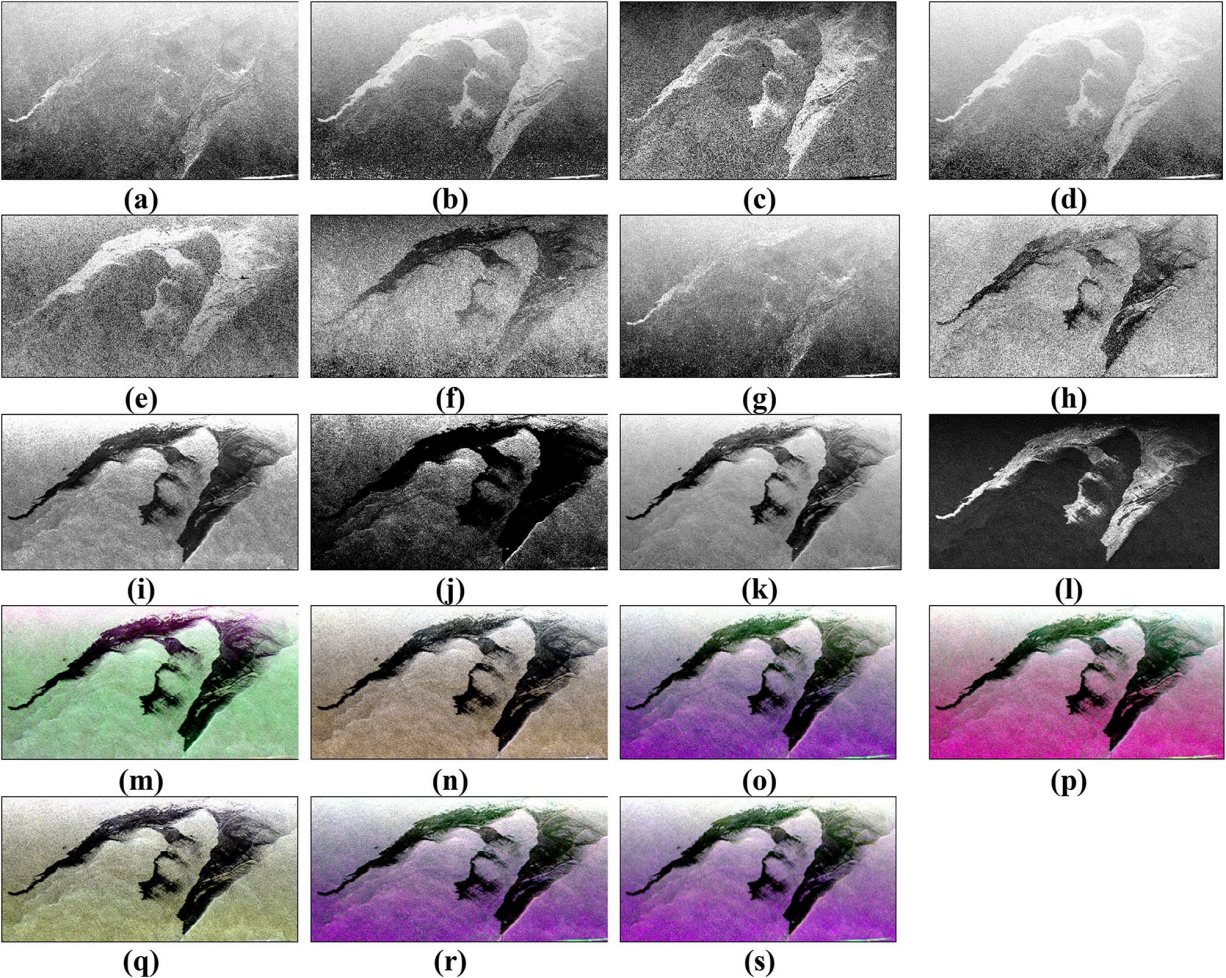
Representation of calculated polarimetric parameters (**a**) Entropy, (**b**) Anisotropy, (**c**) Conformity Coefficient, (**d**) DERD, (**e**) SERD, (**f**) Pedestal Height, (**g**), Scattering Diversity, (**h**) Alpha, (**i**) $\lambda$, (**j**) ${\lambda }_{3}$, (**k**) Shannon Entropy, (**l**) VV Damping ratio, (**m**) Pauli RGB of Coherency matrix, (**n**) LHV, (**o**) LHV Compact-pol decomposition, (**p**) LHV Raney decomposition, (**q**) RHV, (**r**) RHV Compact-pol decomposition and, (**s**) RHV Raney decomposition.

A subset of already radiometrically corrected UAVSAR data is taken and filtered with refined Lee polarimetric speckle filter to minimize the presence of unwanted high-frequency pixels in the image. A window size of 3 × 3 was chosen for polarimetric filtering as a larger window size may suppress the details present in the data. The comparative contrast of the oil spill from water has also enhanced slightly after the speckle filtering. The Lee refined speckle filtered image has been used for further polarimetric parameter derivation. The coherency matrix also has been analyzed for the separability between three components (slick_a_, slick_b,_ and water). All the extracted parameters are shown in Fig. 7. Some parameters like Coherency matrix, RHV Compact-pol, LHV Raney, LHV Compact-pol decomposition, $\lambda$, Shannon entropy seemed to show better contrast between oil and the water body. Other parameters like Anisotropy and Alpha also give good contrast but the oil spill’s signature intensity is not very strong and some parts of the oil spill merge with the water body. Anisotropy and DERD gave a bright oil slick signature in the area where the possibility of having thick oil (more accumulation of oil) layer is high. The Conformity Coefficient seemed to give a fair distinction between water and oil class. However, the separability between water and oil slick was analyzed further from a more statistical point of view in the next section.

### Separability analysis

**Figure 8:**
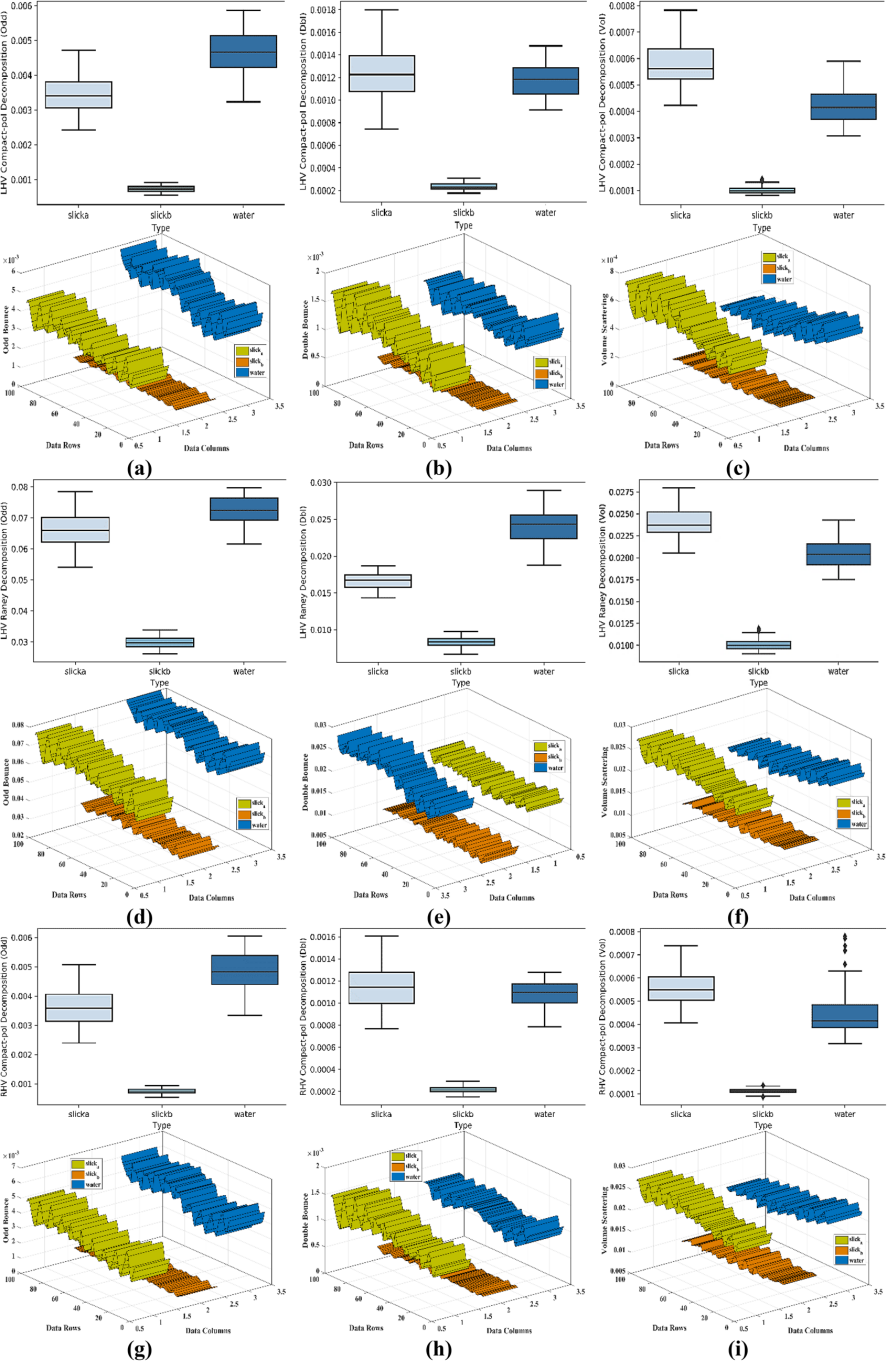

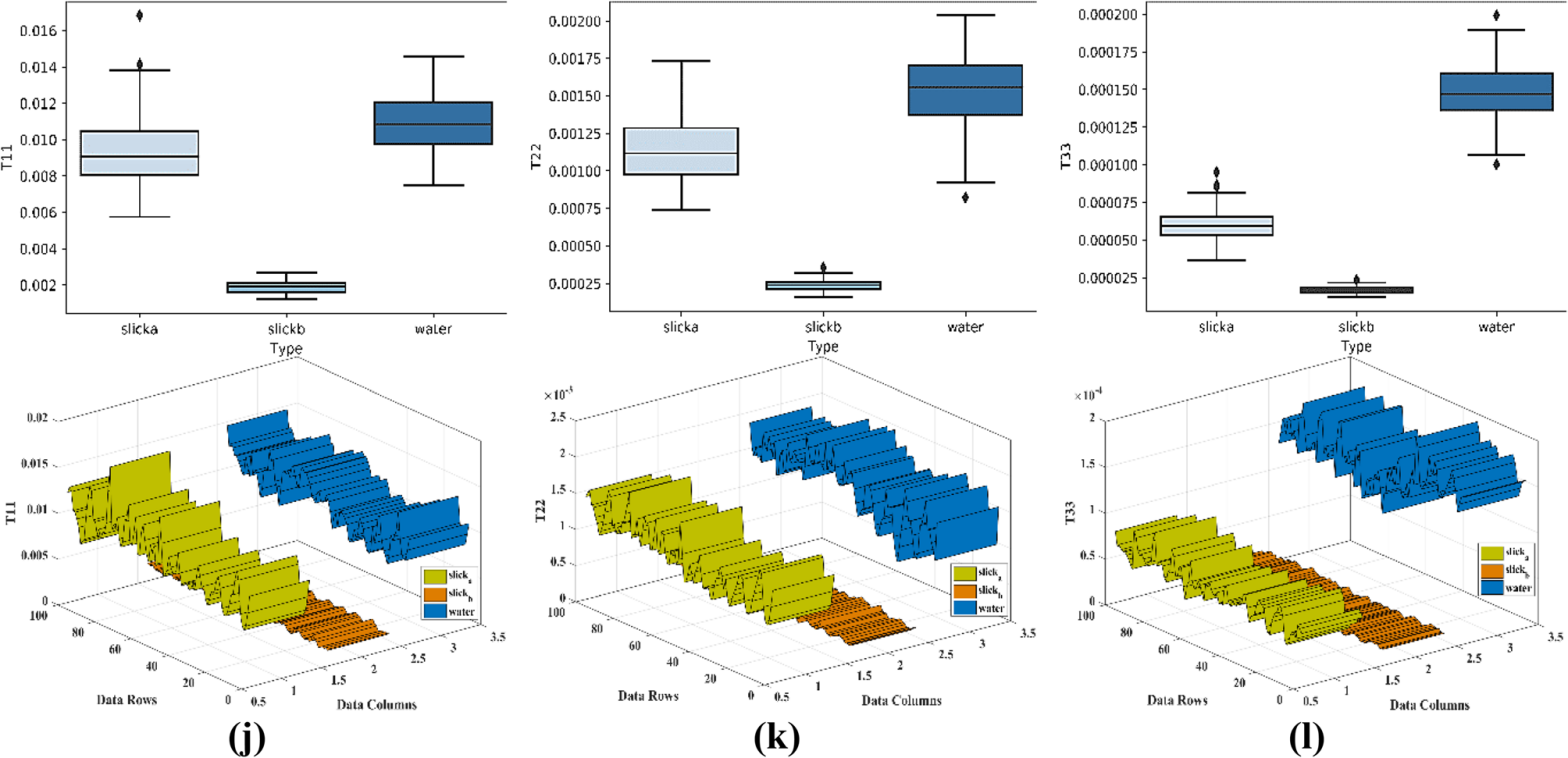
Extracted Box-whiskers and respective separability plots for slick_a_, slick_b_ and water regions for (**a**–**c**) LHV Compact-pol Decomposition, (**d**–**f**) LHV Raney Decomposition, (**g**–**i**) RHV Compact-pol Decomposition and, (**j**–**l**) Coherency Matrix.

For the separability analysis^80^, three ROIs with 100 pixels each were extracted for each parameter. A total of 300 samples for each parameter were used in separability analysis. The box and whiskers plot for each parameter was drawn with one box per dedicated class. The box and whiskers plot is considered reasonably a compact and fairly detailed substitute for a histogram to study the distribution of the data. Each box has a horizontal line within its structure representing the median of the data values. The box plot with the median line lower than the middle position shows the positive skewness while the median line upper than the middle position indicates the negative skewness of the data. In Fig. 8, the box plots for the parameters with the most suitable outcome are shown with their respective three-dimensional separability plots. The boxes in box plots relatively taller than the others show a large degree of variation between the data values within the selected region. On the other side, the compact boxes show a large degree of similarity between the data values of that region. Some boxes have outliers representing the data points that exhibit diverse behavior than the whole group of data. Each separability plot has three ribbons for class slick_a_, slick_b_, and water. Each ribbon shows the pattern of values within the selected ROIs. The ribbon plots of the classes show the separability in a more precise manner. For Entropy, the separability between water and slick_b_ is very minimal. However, the margin between slick_a_-slick_b_ also was very less hence the possibility of the classifier assigning similar values to both the regions becomes very high. Similarly, for Anisotropy, there was a negligible separation between slick_a_- slick_b_, but the separation between slick_a_-slick_b_ and water was acceptable. On the other hand for Alpha, the dissociation observed between water and both slick_a_, slick_b_ was adequate. The values of each class were overlapping with other classes hence a negligible degree of separation was observed between slick_a_-slick_b_. However, a very little separation between water and slick_a_-slick_b_ was observed in SERD. Pedestal height has shown overlapped values between water and slick_a_-slick_b_ elements with outliers in the slick_a_ class. The slick_b_ class in the Pedestal height parameter shows the number of data values (outliers) with the deviation (refer to Table 4 for separability details of each parameter). In Fig. 8, the parameters with maximum separability and optimum results are shown. It is noteworthy that all the parameters shown here, have represented a clear and good separation between the water and slick_b_. Moreover, good separation between slick_a_ and slick_b_ was also achieved by some parameters. However the separability between the water and slick_a_ is not as much as between water- slick_b_ pair in most of the parameters, but adequate to achieve acceptable separation by the classifier. Furthermore, the supervised SVM was applied to the parameters to quantify the separation. The separability for each parameter is summarized in Table 4 for each slick_a_-slick_b_, slick_b_-water, and slick_b_-water pairs against columns Acceptable, Minimum, and Zero/Negligible separability. The selection of Acceptable, Minimum, and Zero/Negligible separation is done purely based on the statistical differences between the data points of respective classes. A check-mark against a parameter shows the retrieved separability for the respective water-slick pair.

### Classification results

**Figure 9:**
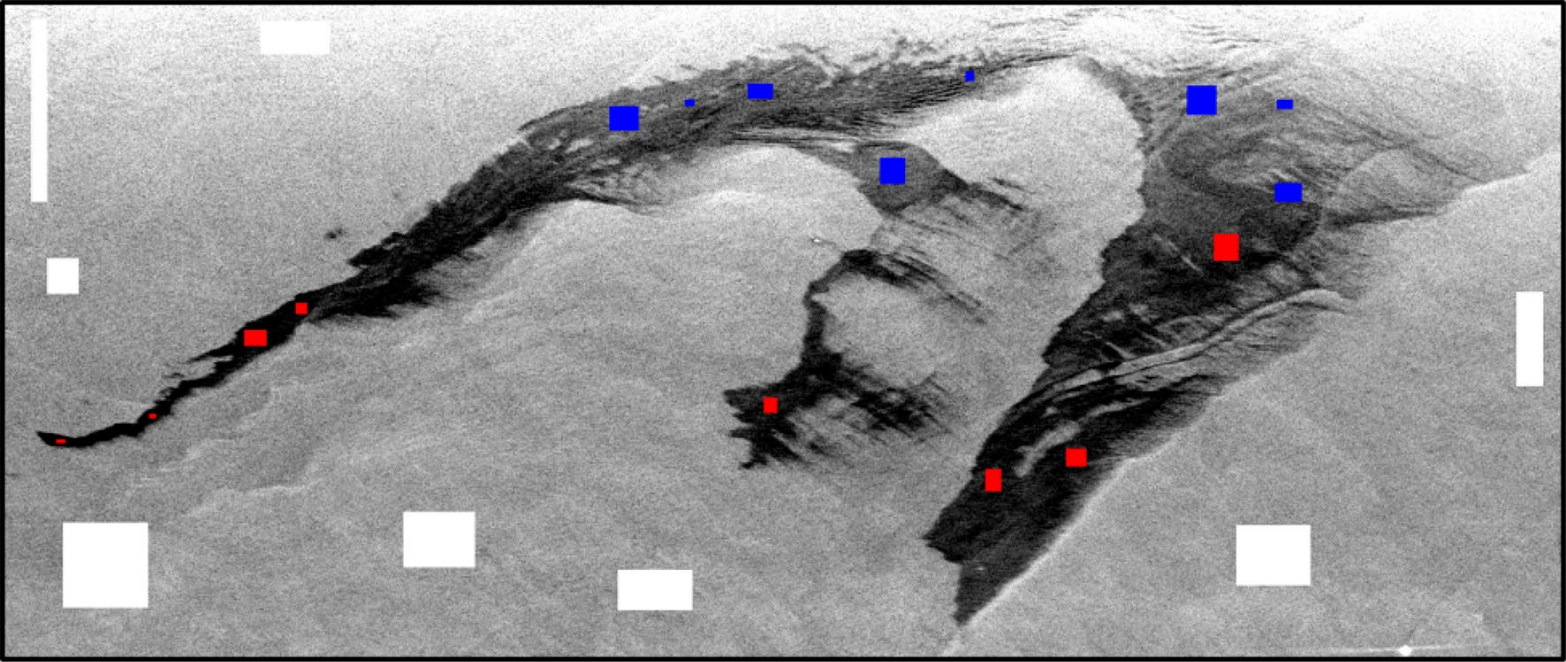
Distribution of training sample for three class over the span image.

Based on the separability analysis, further classification was carried out on the parameters. For the classification, the SVM classifier was utilized. SVM is natively designed for a binary problem by finding a hyperplane between the linearly separable classes. If the classes are not linearly separable then SVM makes use of kernels. Kernels are the measure of similarity or distance between the new data and support vectors. The SVM here is trained with three classes viz., slick_a_, slick_b_, and water. Numerous ROIs were selected for each class having many pixels. The labeling of the classes is done automatically by the classifier during the ROI selection process. The ROIs for three classes were selected over a range and from different regions to make the classifier robust and predict the classification more accurately (see Fig. 9). For each parameter, the ROIs of each class were the same. A total of 8 ROIs were selected for each slick_a_ and slick_b_ and water class. The summary of ROIs is provided in Table 3 which describes the detail of the number of pixels for training and testing used for the SVM classification.

**Table 3:** Training and testing samples for SVM classification.

SVM Classifier classes and number of ROIs	Training pixels (ROI)
slick_a_ (8)	18,603
slick_b_ (8)	11,638
water (8)	150,749

**Figure 10:**
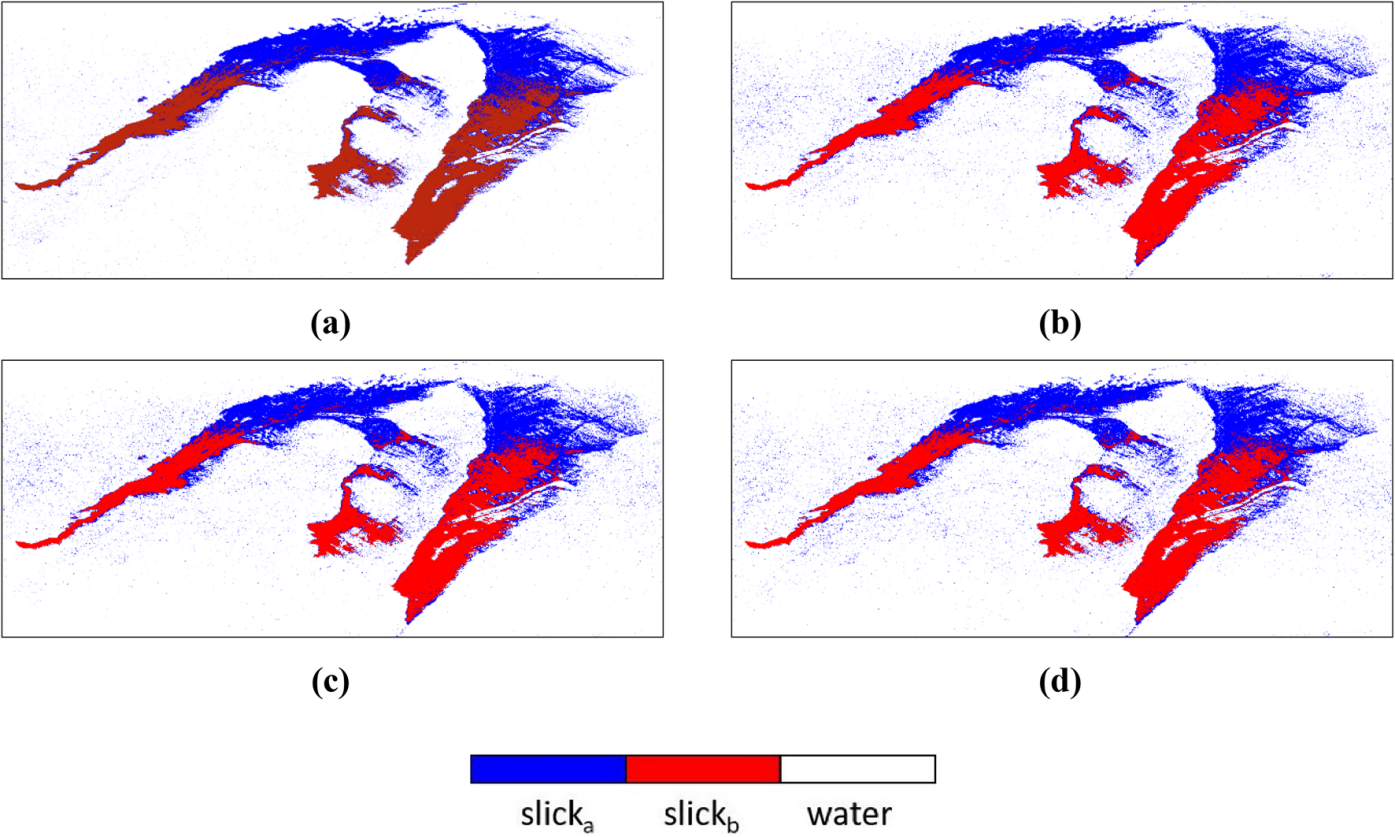
SVM classification results of polarimetric parameters (**a**) Coherency Matrix, (**b**) LHV Compact-pol decomposition, (**c**) LHV Raney decomposition and, (**d**) RHV Compact-pol decomposition.

The most relevant classification outputs are shown in Fig. 10. It was observed that some of the parameters could not be able to separate one oil type from another for example Anisotropy, SERD/DERD, Pedestal Height, and Conformity Coefficient. However, some could not separate the oil from water precisely, even after showing acceptable separability in separability analysis. Parameters like Anisotropy, SERD/DERD, Pedestal height could not separate oil slick regions with possibly different thicknesses (see Table 4). For these parameters, SVM classified the whole region of the oil spill as one (slick_a_ or slick_b_), due to similar backscattering responses from the whole spill region. Another parameter, the conformity coefficient did not perform very well, and the classifier classified the whole slick as one slick type. In the case of $\lambda$, SVM classified the three classes fairly. On the other hand, VV damping ratio also yielded good accuracy, and acceptable separation between two slick_a_ and slick_b_ was seen except at some portions of the image. LHV, RHV, their respective decompositions, and coherency matrix achieved the most accurate predictions between all three classes. Most of the parameters (Alpha, DERD, Entropy, $\lambda$, Pedestal Height, and VV damping ratio) were first tested for gamma value = 0.33. These parameters gave almost similar results when tested with other gamma values (e.g., 0.88, 0.08, 0.55, 0.01, etc.) hence the results are considered for gamma = 0.33 only. LHV Compact-pol decomposition gave accuracy of ~ 98.71% at gamma = 0.33, similar digits were observed for LHV Raney, RHV Compact-pol and RHV Raney decomposition with accuracy of ~ 98.70%, ~ 98.62%, and ~ 98.59% respectively. Coherency matrix has given ~ 99.17% accuracy at gamma = 0.44. The value of gamma decides the range to which the training sample influences the classification. A larger gamma value results in less extent of influence of training set on the classification result or vice versa. Since damping ratio is considered as a standard way to find out the oil slick layer and its relative thickness over water, hence if the classification results are compared with the damping ratio output, it is very evident that SVM has mapped a larger area within the oil slick as thick type (slick_b_). Although, according to damping ratio results (see Fig. 5) the area with more oil accumulation is very lesser than the weathered oil area forming a much thinner layer (slick_b_).

**Table 4:** Achieved separability extent for water-slick pairs against each parameter.

Parameter	Separability								
	Acceptable			Minimum			Negligible		
	Slick_a_-slick_b_	Slick_a_-water	Slick_b_-water	Slick_a_-slick_b_	Slick_a_-water	Slick_b_-water	Slick_a_-slick_b_	Slick_a_-water	Slick_b_-water
Entropy							▲	▲	▲
Anisotropy		▲	▲	▲					
Conformity coefficient					▲	▲	▲		
SERD		▲		▲		▲			
DERD		▲	▲	▲					
Pedestal height		▲		▲		▲			
Scattering diversity					▲	▲	▲		
Alpha		▲				▲	▲		
$\lambda$	▲		▲		▲				
${\lambda }_{3}$	▲	▲	▲						
Shannon entropy	▲		▲		▲				
T11	▲		▲		▲				
T22	▲		▲		▲				
T33	▲	▲	▲						
VV damping ratio	▲		▲		▲				
LHV-C11	▲	▲						▲	
LHV-C22		▲	▲		▲				
LHV Compact-pol-Odd	▲	▲	▲						
LHV Compact-pol-Dbl	▲	▲		▲					
LHV Compact-pol-Vol	▲	▲				▲			
LHV Raney-Odd	▲	▲		▲	▲				
LHV Raney-Dbl	▲	▲				▲			
LHV Raney-Vol	▲	▲				▲			
RHV-C11	▲	▲				▲			
RHV-C22	▲	▲				▲			
RHV compact-pol-Odd	▲	▲	▲						
RHV compact-pol-Dbl	▲		▲			▲			
RHV compact-pol-Vol	▲		▲			▲			
RHV Raney-Odd	▲	▲							
RHV Raney-Dbl	▲	▲		▲					
RHV Raney-Vol	▲	▲				▲			

### Accuracy assessment

The accuracy of any statistical model describes how accurately the finding falls in proximity to the original/field data. The achieved accuracy is highly dependable on the sample selection. The same sample data may give entirely inconsistent outputs for two different parameters. The larger the sample data, the higher are the chances of achieving precise predictions. The achieved accuracy for each of the parameters for all the classes, along with the overall accuracy are described in Table 5. Commission error (or False Positives) gives the stats about the data that is incorrectly classified into the class it doesn’t belong to. Omission error (or False Negatives) calculates the percentage of the data that are of a particular class but wrongly predicted in some other class. Kappa coefficient (K) represents the degree of consensus between the predicted values and the original data. More precisely K estimates the precision level of the classification in comparison to randomly assigned values. K for predicted values x is interpreted as x ≤ 0 (no agreement) to x = 1 (perfect agreement)^81^.

**Table 5:** Achieved accuracy with all tested parameter for each class using SVM classifier.

Parameters		Commission error (%)	Ommission error (%)	Kappa coefficient	Overall accuracy (%)
Entropy	Slick_a_	38.61	39.80	0.38	85.84
	Slick_b_	0	100		
	Water	8.88	3.87		
Anisotropy	Slick_a_	10.76	67.06	0.27	87.87
	Slick_b_	0	100		
	Water	12.17	0.37		
Conformity coefficient	Slick_a_	0	100	0.21	87.02
	Slick_b_	21.75	68.48		
	Water	12.74	0.60		
DERD	Slick_a_	10.94	59.66	0.32	88.39
	Slick_b_	0	100		
	Water	11.63	0.45		
SERD	Slick_a_	11.55	59.37	0.33	88.52
	Slick_b_	0	100		
	Water	11.47	0.33		
Pedestal height	Slick_a_	58.24	34.39	0.35	84.33
	Slick_b_	0	100		
	Water	9.46	7.59		
Scattering diversity	Slick_a_	38.44	31.45	0.44	83.83
	Slick_b_	29.10	89.64		
	Water	12.74	6.42		
Alpha	Slick_a_	0	100	0.39	88.54
	Slick_b_	34.08	42.15		
	Water	10.11	0.79		
$\lambda$	Slick_a_	29.74	70.93	0.79	95.32
	Slick_b_	9.27	4.28		
	Water	4.06	0.44		
${\lambda }_{3}$	Slick_a_	30.03	31.95	0.78	94.45
	Slick_b_	24.70	14.17		
	Water	1.63	2.41		
Shannon entropy	Slick_a_	48.52	99.39	0.58	91.62
	Slick_b_	8.40	2.06		
	Water	8.34	0.25		
Coherency matrix	Slick_a_	0.03	0.03	0.95	99.17
	Slick_b_	0.01	0.03		
	Water	34.18	0.001		
VV damping ratio	Slick_a_	2.44	2.67	0.90	95.98
	Slick_b_	3.85	2.00		
	Water	9.45	9.40		
LHV	Slick_a_	0.08	0.13	0.90	95.98
	Slick_b_	0.02	0.04		
	Water	0.01	0.008		
LHV compact-pol decomposition	Slick_a_	8.00	7.89	0.95	98.71
	Slick_b_	1.14	1.02		
	Water	0.66	0.68		
LHV Raney decomposition	Slick_a_	8.02	7.89	0.95	98.70
	Slick_b_	1.17	0.97		
	Water	0.66	0.69		
RHV	Slick_a_	0.09	0.15	0.90	95.98
	Slick_b_	0.03	0.04		
	Water	0.02	0.009		
RHV compact-pol ecomposition	Slick_a_	9.05	7.81	0.94	98.62
	Slick_b_	1.69	1.58		
	Water	0.62	0.75		
RHV Raney decomposition	Slick_a_	9.63	7.54	0.94	98.59
	Slick_b_	1.63	1.56		
	Water	0.59	0.82		

16 $$K=\frac{P\left(A\right)-P(E)}{1-P(E)}$$

In Eq. (16), $P\left(A\right)$ is the probability of the number of agreement events, whereas $P\left(E\right)$ is the probability of agreement by chance. For LHV and RHV Compact-pol/Raney decomposition, and coherency matrix $K$ is approximately near to 1 representing a strong agreement between the prediction and the original data.

The reason for parameters with high classification accuracy but relatively low $K$ values is a large degree of deviation in predicting the classes accurately. Some parameters like SERD/DERD, anisotropy, and pedestal height, have predicted the whole slick region as one. While parameter, conformity coefficient misclassified a larger portion of the data with relatively good accuracy which is a false prediction. Even after the classification of the whole slick as type one (slick_a_), the accuracy of the classification remains high as the backscattering values are of one type only in some parameters. In other words, the classifier assigned a majority of pixels to a single class resulting in higher accuracy. SVM considered these values and predicted the accuracy against that particular class hence giving high accuracy fractions.

#### Analysis of accuracy

**Figure 11:**
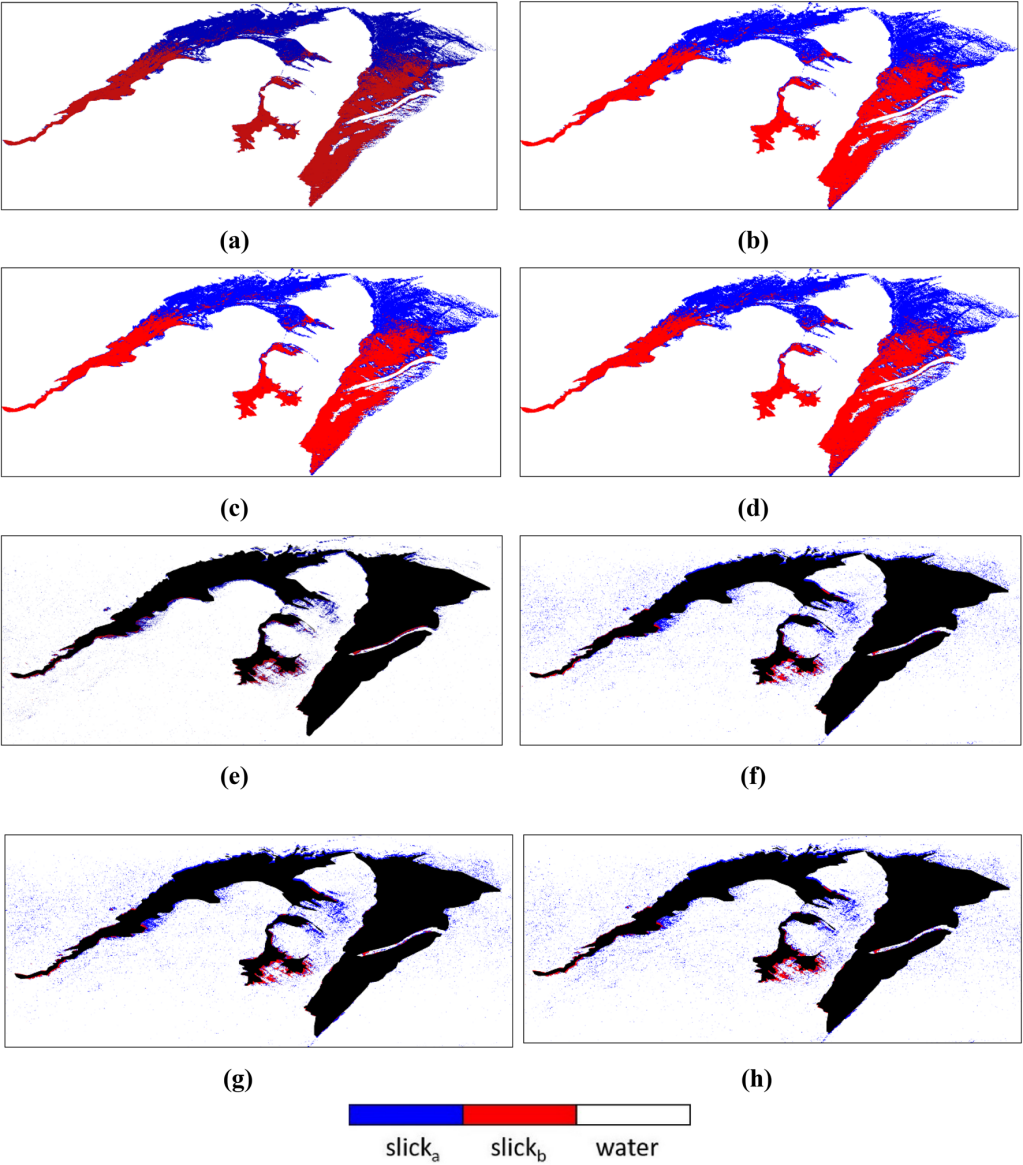
Oil spill subset from (**a**–**d**), and sea region subset from (**e**–**h**) for parameters λ3, Shannon Entropy, Coherency Matrix and, VV damping ratio respectively.

The accuracy of the individual parameter given by the SVM classifier has shown inconsistency. As the extent of accuracy for certain parameters like Entropy, Anisotropy, and Scattering Diversity is very high. It was observed that these parameters have shown very high accuracy without precisely classifying the oil spill and water region. This behavior of SVM is because it misinterpreted a good number of pixels as the wrong class for some parameters. This operation results in SVM thinking that those pixels belong to the wrong class and the classifier calculates the overall accuracy based on these assumptions. To overcome this problem, a pixel-based accuracy assessment approach is applied to the classification outputs. A precise subset of the oil spill was extracted from each classification output but only parameters with relevant outputs were included. A subset only containing the sea area was also extracted from each parameter to know the accuracy of the classifier in mapping the water correctly (see Fig. 11). The white portion in Fig. 11a–d represents the masked out sea area and only the oil spill area is taken in the subset. This will help in correctly calculating the number of pixels assigned to different classes. Similarly, in Fig. 11e–h the black portion represents the masked out oil spill zone to correctly calculate the misclassified and correctly classified sea area pixel.

The pixel-based accuracy assessment was done with the following steps:

1. Extract the oil spill and sea area subset separately for each parameter.
2. Calculate the extent of correctly classified pixels for the oil spill and the sea region.
3. Calculate the extent of misclassification in both oil and water class.
4. Estimate the overall accuracy of the classifier for both the oil and water class.

**Figure 12:**
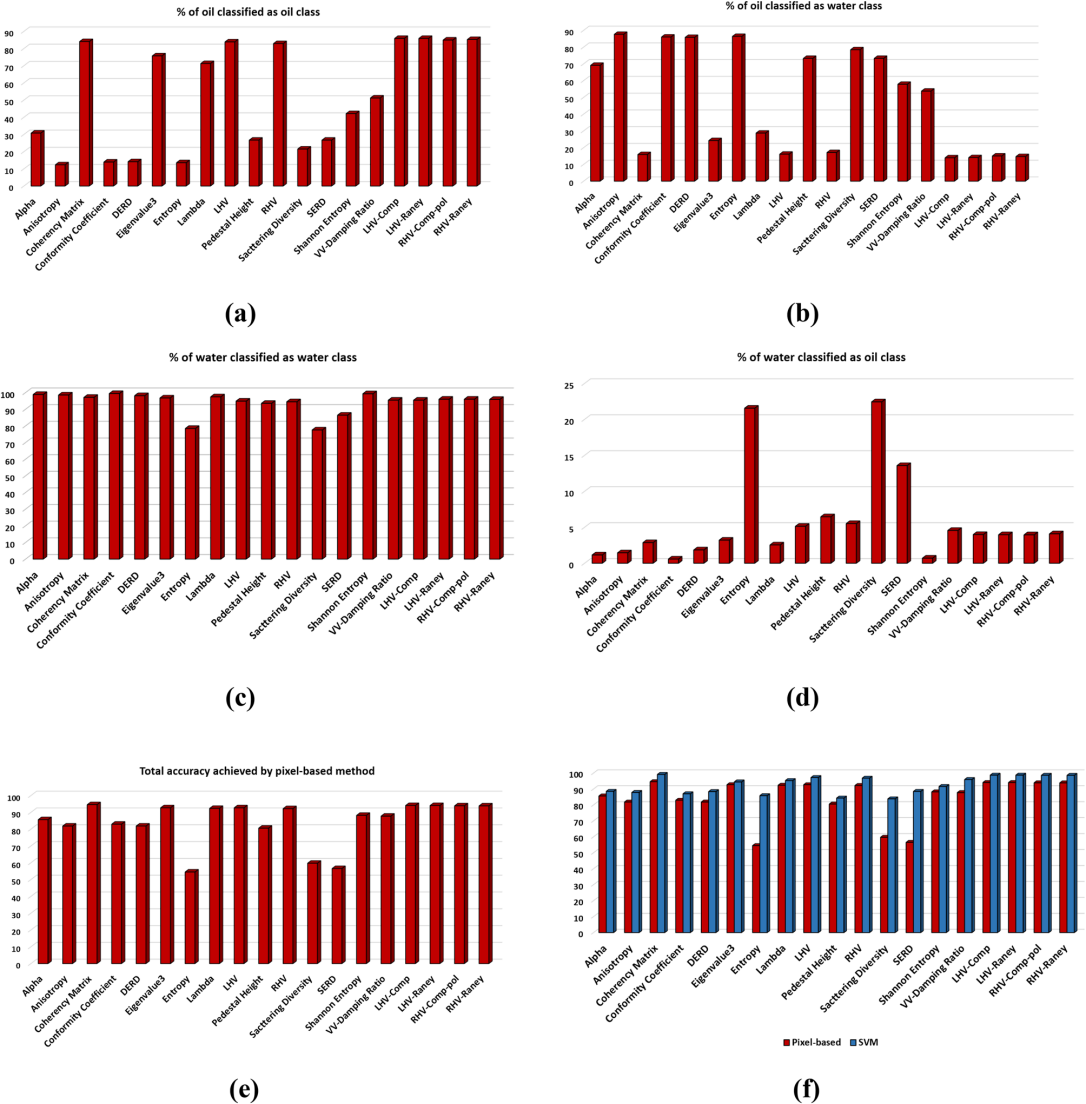
The bar graphs describe the percentage of (**a**) correctly classified oil spill area in the oil spill subset, (**b**) oil spill misclassified as water in oil spill subset, (**c**) correctly classified water area in the sea subset, (**d**) water area misclassified as oil in the sea subset, (**e**) overall accuracy of oil spill + water, (**f**) comparison of calculated pixel-based accuracy with the SVM accuracy.

In Fig. 12a, coherency matrix and LHV decomposition show the highest percentage of the correctly classified oil region. Anisotropy parameter in Fig. 12b has registered the maximum percentage of oil spill misclassified as water. In Fig. 12c, the maximum number of parameters has performed well in classifying water correctly except Entropy and Scattering Diversity which have shown comparatively more misclassification behavior. This behavior by these parameters is proved by the statistics given in Fig. 12d, where Entropy and Scattering Diversity have shown maximum misclassification percentage. Here, it was also observed that Conformity Coefficient and Shannon Entropy has provided a relatively very low misclassification degree with only ~ 0.58% and ~ 0.70% misclassification respectively. The overall accuracy (see Fig. 12e) is quite high in values for the parameters that did not perform very well in fittingly mapping the oil spill. The reason behind this is the classification accuracy for the sea region in many parameters was very good. However, these parameters did not perform well in the successful mapping of the oil spill and ended in yielding a good degree of misclassification. Furthermore, while considering the overall accuracy, both the sea and oil spill were considered and the large accuracy values of sea region classification compensated for the low accuracy of the oil spill classification.

The acquired accuracy for each parameter is shown in Table 6 using a pixel-based method. The comparison is done to know the extent of misclassification given by SVM. A significant decrement in accuracy percentage can be seen with the pixel-based method resulting in more accurate outputs. Hence, LHV/RHV decompositions, as well as the coherency matrix, were more successful in oil spill classification than the other tested parameters, but if the overall accuracy is considered, except Alpha and Conformity Coefficient, most of the parameters gave good accuracy. It was observed that most of the parameters shown a significant fall in accuracy when compared to SVM accuracy (see Fig. 12f), majorly in Entropy, Scattering Diversity, and SERD. Other parameters have also shown a certain level of decrement in the values but it was not as significant as these three parameters. The reason behind the high accuracy of parameters in SVM may lie in its functioning.

**Table 6:** Achieved accuracy with all tested parameter for each class using SVM classifier and the pixel-based method.

Parameter	Accuracy (%)	
	SVM	Pixel-based
Entropy	85.84	54.52
Anisotropy	87.87	81.85
Conformity coefficient	87.02	82.97
DERD	88.39	81.88
SERD	88.52	56.57
Pedestal height	84.33	80.60
Scattering diversity	83.83	59.75
Alpha	88.54	85.67
$\lambda$	95.32	92.38
${\lambda }_{3}$	94.45	92.74
Shannon entropy	91.62	88.22
Coherency matrix	99.17	94.60
VV damping ratio	95.98	87.76
LHV	97.22	92.72
LHV-Raney	98.70	94.09
LHV-Comp-pol	98.71	94.09
RHV	96.82	92.23
RHV-Raney	98.59	93.87
RHV-Comp-pol	98.62	93.91

According to the statistic outcomes, SVM falls into the tendency of giving high accuracy even after misclassification as for SVM the classified data belongs to a certain class and it has been mapped almost correctly which ultimately justifies high accuracy values. The study is conducted on an oil slick captured by an L-band UAVSAR sensor. The following methodology was successful in achieving good results with a capability of discriminating between water–oil and oil-oil depending on thickness or backscattering variation. The overall complexity of the algorithm in terms of processing and storage is the image size and underlying machine architecture-dependent. The overall methodology works quickly and fast except for the classification part, which takes a little bit more time. The classification algorithm is a supervised machine learning approach and highly dependable on the training sample size. The computational time increase with the number and size of training samples. The basis of SVM is statistical theory and it generally provides the good ability of generalization with a limited number of training samples. Hence, the computational burden is subject to the image size/resolution and the number/size of training samples.

It also depends on the type of kernel function and the regularization parameter (C). Adjusting these parameters can have a significant effect on the computational as well as the time complexity of the classifier.

### Implementation of the methodology on DATA2

#### Separability analysis and classification results

**Figure 13:**
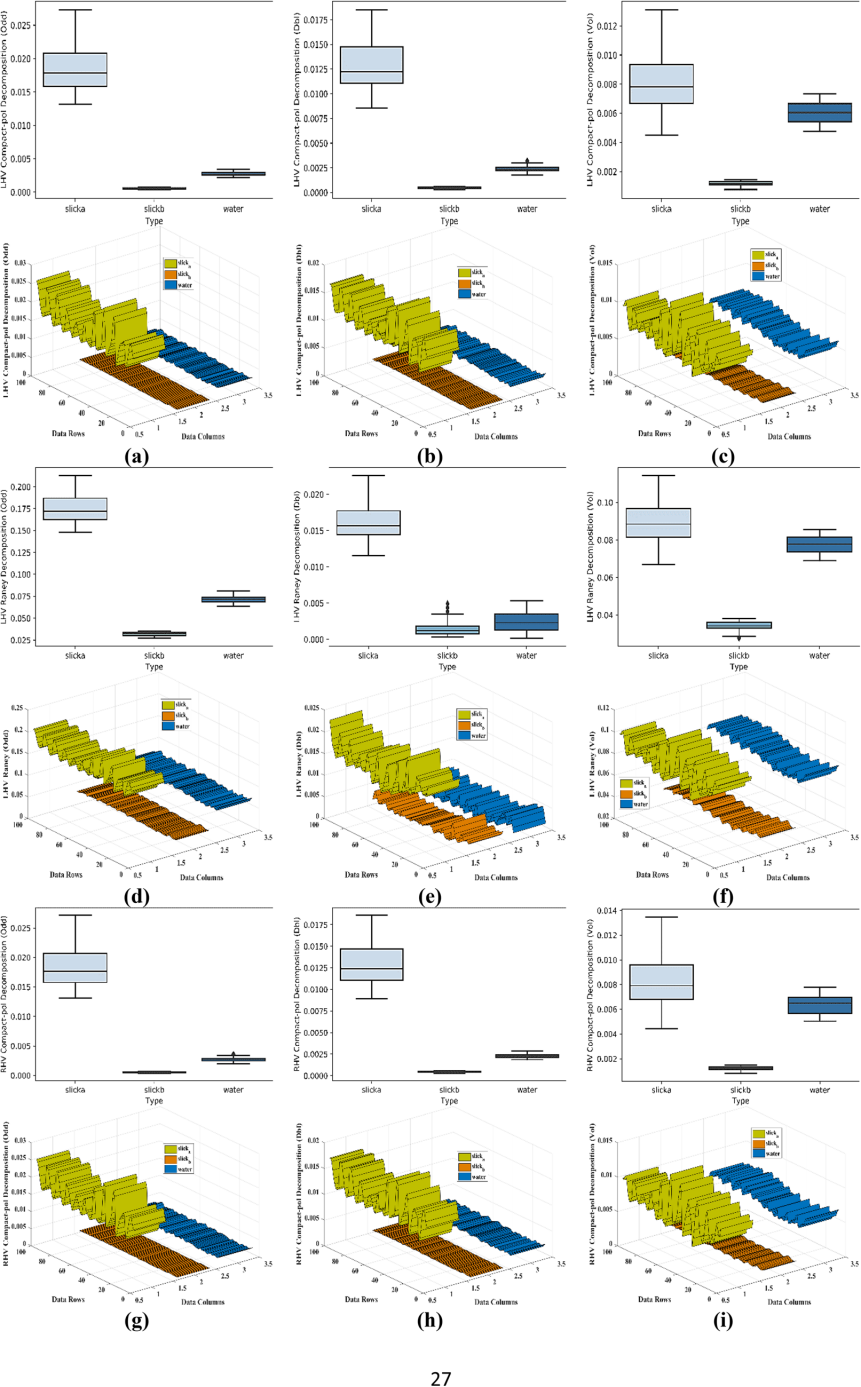

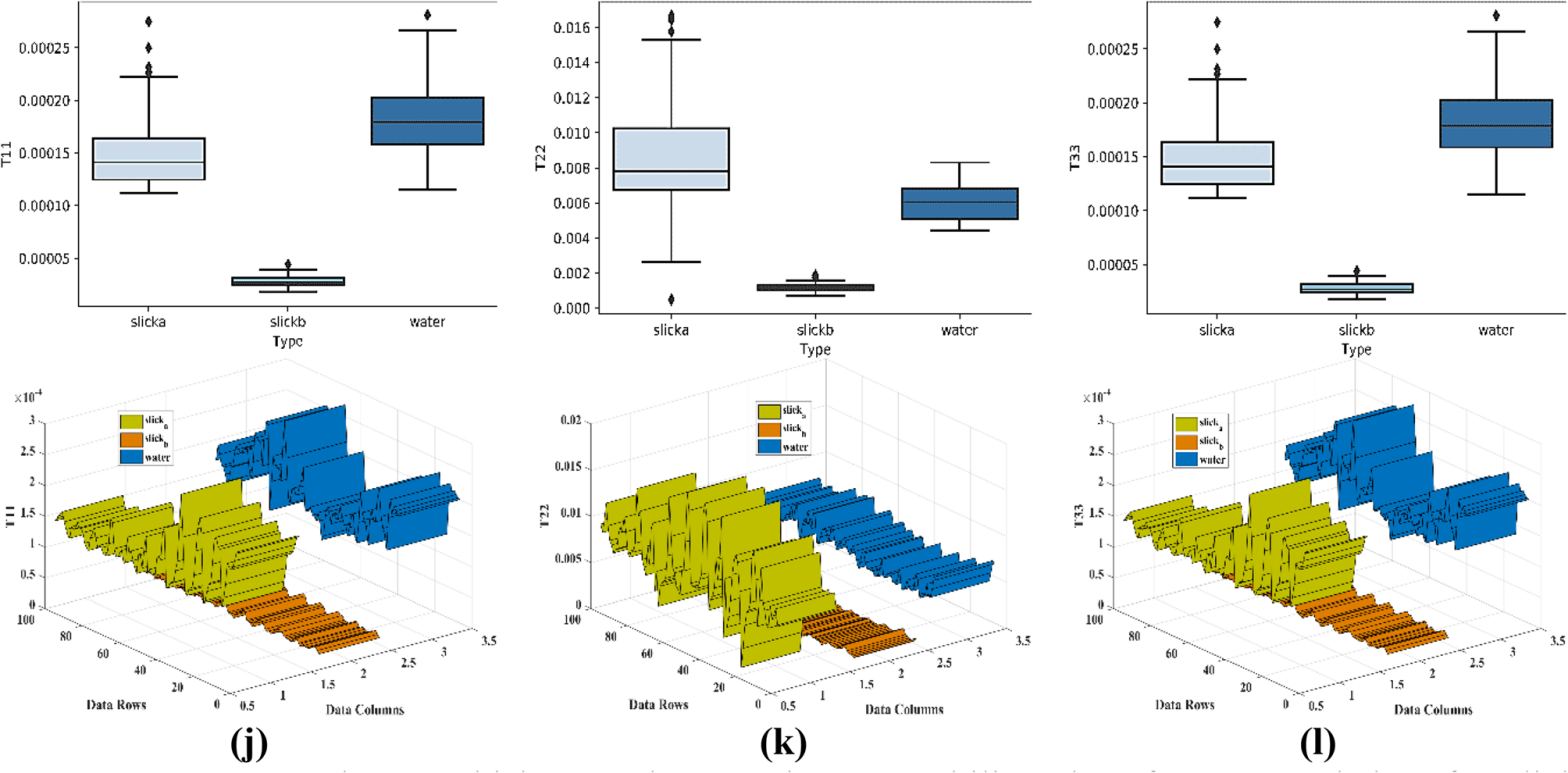
Extracted Box-whiskers and respective separability plots from second data for slicka, slickb and water regions for (**a**–**c**) LHV Compact-pol Decomposition, (**d**–**f**) LHV Raney Decomposition, (**g**–**i**) RHV Compact-pol Decomposition and, (**j**–**l**) Coherency Matrix.

The proposed methodology has been implemented to DATA2 to know the effectiveness of the overall procedure. The trained SVM model (on DATA1) is tested on DATA2 for all the parameters, out of which only the parameters that have shown good accuracy are included. It is noteworthy that the parameters that have shown good accuracy for DATA1 have shown good accuracy for DATA 2 also. The following sections include the separability and classification results of the most parameters with higher accuracy. These parameters are LHV Compact-pol Decomposition, LHV Raney Decomposition, RHV Compact-pol Decomposition, and Coherency matrix. The Box plot along with the separability plot for each has been provided in Fig. 13. In all the parameters slick_a_ has shown very low data values compare to slick_b_ and this difference resulted in good separability between slick_a_ and slick_b_. However, water class values merge with slick_a_/slick_b_ class in some parameters, and this explains the misclassification of the sea class as one of the oil classes.

**Figure 14:**
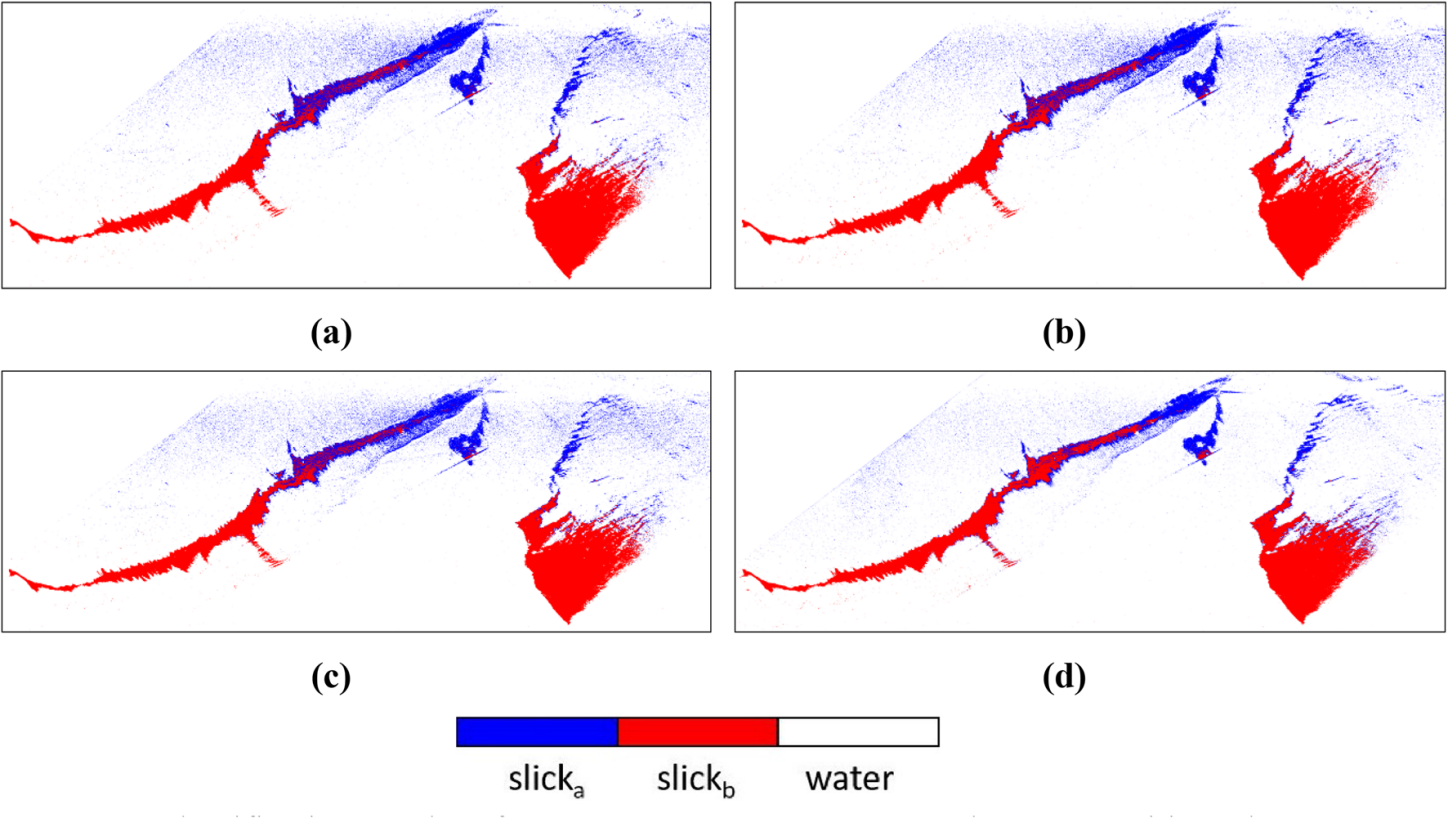
Classification results of DATA2 (**a**) LHV Compact-pol Decomposition, (**b**) LHV Raney Decomposition, (**c**) RHV Compact-pol Decomposition, and (**d**) Coherency Matrix.

The classification maps of the parameters that shown good accuracy are represented in Fig. 14. A subset of the data was extracted by masking out the land present in the scene. The misclassification of the sea class is more prominent in the upper side of the data as the backscattering values of the sea and slick_a_ classes were very similar as seen in the box and separability plots in Fig. 13. However, the classifier mapped the edges of the slick very well in the upper zone as the chances of oil having varying thickness were prominent there.

#### Accuracy assessment

The accuracy assessment of the overall method has been done similar to DATA1. The table for the parameters has been provided along with the accuracy values yielded by SVM and the accuracy values estimated by the pixel-based method (see Table 7). In the case of DATA2, it was observed that the variation between SVM and pixel-based accuracy is very less.

**Table 7:** Accuracy comparison for DATA2 parameters.

Parameter	Accuracy (%)	
	SVM	Pixel-based
Coherency Matrix	96.28	96.97
LHV-Raney	96.03	96.04
LHV-Comp-pol	96.19	95.94
RHV-Comp-pol	95.48	95.89

It can be seen from Table 7, that the accuracy values are very close, and there is not much variation. In the case of LHV-Comp-pol, a decrement of less than 1% is seen in overall accuracy. Moreover, in this case, the accuracy estimated by the pixel-based method seems to be in good agreement with SVM accuracy which indicates its effectiveness. This analysis demonstrates the necessity of the analysis of accuracy estimated by SVM classifier. After analyzing the accuracy results the under/overestimation of the accuracy can be known effectively, making the interpretation of results more robust.

## Conclusions

Since the past couple of years, polarimetric SAR images have provided reliable results in monitoring oil spills. The detection of oil spills has become very feasible with growing techniques. However, the detection part is not the difficult phase, the difficulty lies in distinguishing between the oil slicks zones (potentially varying oil thickness areas). This study was focused on segmenting the oil slick into the two regions based on their backscattering responses. An attempt is made to simulate the compact-pol data from the quad-pol data to explore the possibility of utilizing both the polarimetric modes with the help of only one dataset for the oil slick discrimination. The experimented results on two datasets have shown the supremacy of the outputs from the compact-pol data over quad-pol data results with slightly higher accuracy. A new index using VV damping ratio and CPR was also investigated to achieve a better contrast between oil slick and water. A significant increment of the contrast between water and the oil slick was observed with the tested index. However, the full potential of this index can be further analyzed. This study utilized 19 parameters which were tested using a common approach. The outcomes of all parameters diversified widely. The selection of samples for the separability analysis is consistent for each parameter and the same approach was done for the classification procedure. In the separability analysis, the parameters tend to show minimum separability for the chosen ROIs but on the contrary, the same parameters may show good separability for a completely different set of ROIs. Some analyzed parameters were not able to distinguish between two classes (one oil type from another) even after showing an acceptable extent of separability. This can be concluded as the separability analysis and the classification methods are training set dependent approaches. The classification outcomes were further analyzed for the correct extent of accuracy. A pixel-based accuracy analysis approach has shown significant decrement in the yielded SVM accuracy in DATA1 and good agreement in the case of DATA2. It was observed that for basic oil-to-oil discrimination, VV damping ratio, coherency matrix, and LHV/RHV components solely provided better results. Some parameters failed to achieve an acceptable classification accuracy even after giving appropriate separability, while other parameters did provide some reliable results. Although the VV-damping ratio has also shown a good confirmation of the oil accumulation in some areas and some parameters gave very close results to that. These possibilities can further be analyzed for better understanding. A combination of two or more classifiers can also be utilized for more clear outcomes with defined slick and water boundaries but the outcome of the same parameter with different underlying conditions may differ.

## Supplementary Information

Supplementary Information
