# Supplementary material for: Dark spot detection for characterization of marine surface slicks using UAVSAR quad-pol data
Source: Sci Rep. 2021 Apr 26;11:8975. doi: 10.1038/s41598-021-88301-9 (PMC8076205; doi:10.1038/s41598-021-88301-9)
Supplement: Supplementary file 1 — Supplementary Information [file 41598_2021_88301_MOESM1_ESM.docx]

Dark Spot Detection for Characterization of Marine Surface Slicks using UAVSAR Quad-pol Data

Vaishali Chaudhary a, Shashi Kumar a,*

^a^Photogrammetry & Remote Sensing Department, Indian Institute of Remote Sensing (ISRO), India

*****Correspondence: [shashi@iirs.gov.in](mailto:shashi@iirs.gov.in)

# Appendix

# A.1 Incidence angle correction

|  | $\sigma_{ref}^{o}= \frac{\sigma_{\theta_{i}}^{o}\cos^{n} \left( \theta_{ref} \right)}{\cos^{n}\left( \theta_{i} \right)}$ | A-1 |
| --- | --- | --- |

The change in radiation is cosine dependent function. Hence the normalization is at an angle $\theta_{\mathrm{ref}}$ for the respective radar backscatter, $\sigma_{0}$ and incidence angle $\theta_{i}$ is given in Equation A-1 as:

Where, $\sigma_{\theta_{i}}^{o}$ is the radar backscatter response at $\theta_{i}$, $\theta_{ref}$ is the reference incidence angle at which the normalization is done, and $n$is the roughness dependent parameter. Note that $\sigma_{ref}^{o}$ is an incidence angle independent parameter. The value of $n$ for each channel is calculated through linear regression as given in Equation A-2.

|  | $y=an+b$ | A-2 |
| --- | --- | --- |

The value of $n$ for UAVSAR datafiles ranged between 1.0693 and 6.2647. These values were used with the reference angle $\theta_{ref}=43.42636$ (mean value of ${21.45}^{o}$ to ${65.37}^{o}$). To know the effectiveness of the incidence angle normalization procedure, a transect plot was plotted (see Figure A1) at column no 2922 for approximately 1200 range pixels covering the part of the oil slick region (see Figure 4a). From Figure 4b, a visual significant change in radar backscatter can be observed. It can be observed from the plot in Figure A1 that the overall backscattering response has been normalized after the incidence angle correction. The data image had a strong antenna pattern before the correction from near to far range, which seems minimized up to an acceptable extent in the after incidence angle correction image.


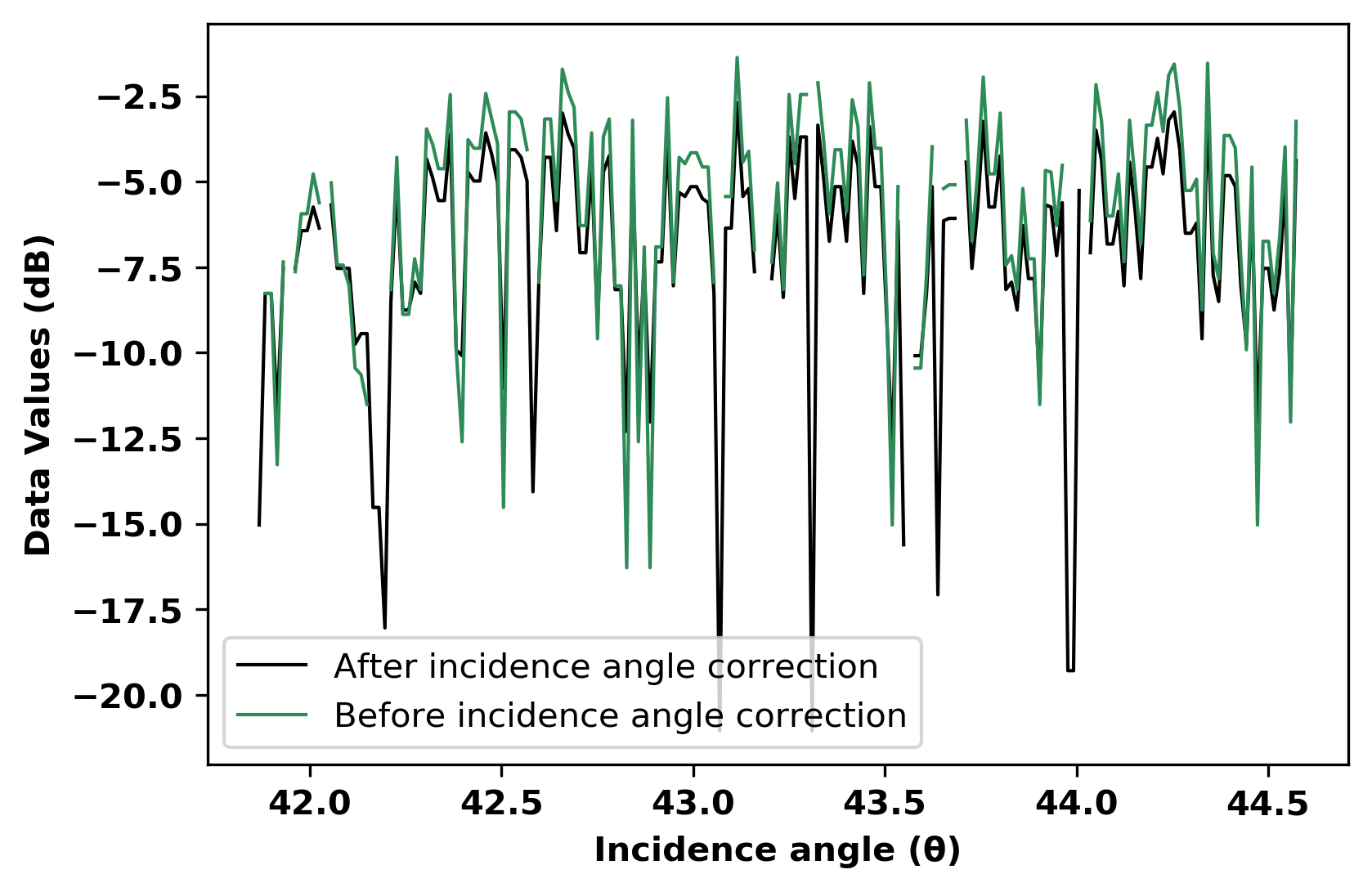


**Figure A1:** Variation of radar backscattering before and after incidence angle correction
